# The impact of the tumor microenvironment on the survival of penile cancer patients

**DOI:** 10.1038/s41598-024-70855-z

**Published:** 2024-09-27

**Authors:** Stefan Lohse, Jan Niklas Mink, Lea Eckhart, Muriel Charlotte Hans, Leuart Jusufi, Anabel Zwick, Tobias Mohr, Isabelle Ariane Bley, Oybek Khalmurzaev, Vsevolod Borisovich Matveev, Philine Loertzer, Alexey Pryalukhin, Arndt Hartmann, Carol-Immanuel Geppert, Hagen Loertzer, Heiko Wunderlich, Hans-Peter Lenhof, Carsten Maik Naumann, Holger Kalthoff, Kerstin Junker

**Affiliations:** 1 Institute for Virology, Saarland University Medical Center, Saarland University, Kirrberger Str. Building 47, 66421 Homburg, Germany; 2 Department of Urology and Pediatric Urology, Saarland University, 66421 Homburg, Germany; 3 Center for Bioinformatics, Saarland Informatics Campus, Saarland University, Saarbrücken, Germany; 4 Department of Urology, Federal State Budgetary Institution “N.N. Blokhin National Medical Research Center of Oncology”, Ministry of Health of the Russian Federation, Moscow, 115478 Russian Federation; 5 Department of Urology and Pediatric Urology, Westpfalz-Klinikum, Kaiserslautern, Germany; 6 Institute of Pathology, Saarland University Medical Centre, 66421 Homburg, Germany; 7 Institute of Pathology, University Erlangen-Nuremberg, 91054 Erlangen, Germany; 8 Clinic of Urology and Pediatric Urology, St. Georg Klinikum, 99817 Eisenach, Germany; 9 Department of Urology and Pediatric Urology, University Hospital Schleswig Holstein, Kiel, Germany; 10 Institute for Experimental Cancer Research, Medical Faculty, Christian Albrecht University, Kiel, Germany; 11 Present Address: Current Address: Leibniz-Institute for New Materials (INM), Campus D2.2, 66123 Saarbrücken, Germany

**Keywords:** Penile cancer, HPV, Neutrophils, Survival, CD147, Tumor microenvironment, Penile cancer, Cancer microenvironment

## Abstract

PeCa is a rare entity with rising incidence rates due to increased infections with human papillomaviruses (HPV). The distinct subtypes of PeCa with an individual pathogenesis demand biomarkers for a precise patient risk assessment regarding disease progression and therapeutic susceptibility. We recently identified promising candidates associated with an HPV-instructed tumor microenvironment (TME) using HPV-positive PeCa cell lines and tissue microarrays (TMA). The capacity of HPV + p63 + PeCa cells to release neutrophil-attracting CXCL-8 provided a molecular link explaining the infiltration of CD15 + myeloid cells in PeCa specimens. The candidate biomarkers HPV, p63, CD15, DKK1, and CD147 linked a tumor-promoting TME with a higher TNM classification reflecting more aggressive and metastasizing cancers. Based on immune-reactive scores (IRS) from TMA staining for these biomarkers, we calculated correlations and conducted association analyses to assess the degree of relationship between all biomarkers. We then conducted Kaplan–Meier survival estimates and Cox regression analyses to delineate the impact on PeCa patient survival. There is a notable predictive potential regarding the survival of patients with biomarker profiles beyond the potency of the individual biomarker. From all candidate biomarkers and biomarker profiles, the combination of CD147 and infiltrating CD15 + cells linked to an active HPV-driven transformation displayed cancer-immune dynamics with dismal prognosis for patients. After deciphering relevant interdependencies, the HPV + CD147 + CD15 + status was the most potent profile predicting metastasis-free survival of PeCa patients. The results of this report underscore the need for analysis of the TME and the development of multi-parameter composite scores that reflect fundamental cancer-immune relationships to tailor therapeutic interventions based on actual cancer immune dynamics.

## Introduction

The reorganization of the TME towards a tumor-promoting environment composed of different supporting cellular species belongs to the hallmarks of cancer^1,2^. The TME comprises cellular and non-cellular elements surrounding and embedding tumor cells including the extracellular matrix, a specific cytokine configuration and different types of immune, stromal, and vascular cells. Heterogeneity, angiogenesis, immunosuppression, and a certain level of cellular plasticity regarding phenotypes and functional properties contribute to the TMEs complexity and influence tumor development and response to treatment. Compromised immune and stromal cells named tumor-associated neutrophils (TAN), myeloid-derived suppressor cells (MDSC), and cancer-associated fibroblasts (CAF) support disease progression at multiple steps and modes of action^2,3^. The patients’ TME phenotype predicts immunotherapeutic susceptibility and immune evasion and provides metastasis-associated signatures^3–5^. Cancer and its progression are a systemic issue involving multiple parameters, environments, and cell populations that support tumoral and metastatic niches^3^. An increased understanding of the individual pathogenesis underlying nature will thus help to stratify patients according to their risk for metastases, survival prognosis, and therapeutic response.

Penile cancer (PeCa) is a rare disease presenting with individual subtypes and pathogeneses^6^. The incidence rates from 0.1/100,000 men in European countries to 6.15/100,000 in Brazil reflect considerable geographical differences^7,8^. Among classical inducers such as recurrent inflammation, smoking, and poor hygiene, recurrent infections with high-risk variants of HPV are a risk factor of rising importance^6,8^. PeCa and penile intraepithelial neoplasia were substantially associated with HPV infection^7^. A recent meta-analysis delineated the pooled prevalence of HPV DNA in PeCa as 50.8%^7^. The high-risk type HPV16 was the predominant type with 68.3%^7^. Of those 79.6% were positive for p16INK4A, a surrogate marker for an active HPV-driven transformation^7^. The two viral oncoproteins E6 and E7 mediate the HPV-driven transformation of high-risk HPV types such as HPV16, decouple the cell cycle from its regulation by degrading p53, pRB, and cause an elevated expression of p16INK4A^9^. The HPV status, defined by positive PCR and p16INK4A staining, may have a predictive value but this is still under debate^10^. Markers reflecting the tumor biology and the tumor-promoting nature of the TME in addition to the HPV status may help to provide a clearer picture and more precise prognostic estimation.

Our recent reports based on HPV-positive PeCa cell lines and TMA containing clinical PeCa specimens underlined the relevance of TME reprogramming in HPV + PeCa specimens^11–13^. We identified mechanisms and putative biomarkers beyond the HPV status, such as p63, CXCL-8, CD15, DKK1, and CD147, with the potential to mark patients at higher risk for disease progression and lymph node metastases. Moreover, the CXCL-8-rich conditioned media of PeCa cell lines with high viral oncoprotein expression facilitated their susceptibility towards IgA antibody dependent neutrophil-mediated tumor cell killing, while the membranous expression of CD147 seemed to diminish it^11,13^. These data suggested a successive reprogramming of the TME in HPV + PeCa specimens towards ongoing chemotaxis of neutrophils and their polarization into TAN or neutrophil-derived suppressor cells (NDSC). The modulation of the immune system and the immune cell infiltrate composition, with neutrophils as a core component, display hallmarks of PeCa and head and neck cancer, two HPV-associated entities^3,14,15^. An increase in NDSC is a critical characteristic of non-responders to chemotherapeutic or T-cell-centered immunotherapeutic interventions^16–18^. Data on cervical cancer, a majorly HPV-driven entity, further support this observation^19,20^. Thus, data from different HPV-associated entities provide mounting evidence that patients with myeloid cells involved in carcinogenesis are at high risk for disease progression, poor survival, and reduced therapeutic response.

Considering the current landscape, the absence of any established biomarkers recommended for PeCa diagnostics underscores the need for promising candidates that facilitate risk assessment and tailor therapeutic decisions. Our previous investigations have delineated potential biomarkers, extending beyond the realm of HPV status, which encompass p63, CD15, DKK1 and CD147^11–13^. These candidates were elaborated using unique novel HPV-positive PeCa cell lines and TMA with linked clinical data to the contained PeCa specimens^11–13,21^. These TMA were stained using immunohistochemistry and evaluated by IRS. The results associated a notable potential to predict aggressive growing metastasizing cancers with the combinations HPV + p63 + CD15, HPV + DKK1 and HPV + CD147 + CD15^11–13^. In total, these findings point towards a scenario where HPV-positive PeCa cells orchestrate the recruitment of neutrophils into a TME at the tumor center fostering their polarization into TAN^11–13^. Here, by calculating Kaplan–Meier survival estimates and Cox regressions we delved into the prognostic implications of these parameters and unveiled promising candidates, underscoring the requirement for biomarkers to reflect the intricacies of tumor biology beyond the HPV status.

## Results

### Correlation, similarity, and association analyses specified the relationships between candidate TME-related biomarkers

We previously evaluated potential biomarkers (HPV, p63, CD15, DKK1, CD147) using novel HPV-positive PeCa cell lines and TMA with matching clinic-pathological data for their association with invasive and metastasizing cancers^11–13^. The current state of the art^11–13,22,23^ suggested a dependency between the individual parameters^11–13^. Briefly, HPV can induce p63, CD147, and, as part of a negative feedback loop, DKK1, while the transcription factor p63 can induce CXCL8. The latter and CD147 mediate the immigration of CD15 cells with DKK1 supporting the intra-tumoral TAN burden^12^.

To address the level of dependency we calculated Pearson (linear relationships) and Distance (linear and non-linear relationships) correlation, and Hoeffding’s testing of interdependence based on the previously published IRS^11–13^ (Table 1) comparing all pairs of parameters (HPV, p63, CD15, DKK1, CD147). The pairs HPV + DKK1, followed by HPV + CD15 and CD15 + p63, had the highest Pearson and Distance correlation coefficients suggesting associations. However, from a numerical perspective, neither Pearson nor Distance correlation nor Hoeffding’s testing supported strong dependencies.

**Table 1:** Correlation and association analyses with schematic illustration of potential relationships.

P1	P2	Pearson_correlation	Distance_correlation	Hoeffding_Pvalue	Hoeffding_Pvalue_Bon
Calculation based on IRS (0–12)					
DKK1	HPV	0.3343	0.3223	0.2100	1.0000
CD15	HPV	0.2647	0.2726	0.4342	1.0000
DKK1	CD15	-0.0769	0.1467	1.0000	1.0000
p63	CD15	0.2315	0.2309	0.4508	1.0000
p63	CD147	0.2239	0.2194	0.6575	1.0000
CD147	HPV	0.1114	0.1453	1.0000	1.0000
p63	DKK1	0.1344	0.1607	0.9568	1.0000
p63	HPV	-0.0282	0.1271	1.0000	1.0000
CD147	DKK1	-0.0686	0.1447	1.0000	1.0000
CD147	CD15	-0.0453	0.1430	1.0000	1.0000

P1	P2	Fishers_exact_Pvalue	Fishers_exact_Pvalue_Bon		
Binarized IRS-based calculations (IRS 0–2 = negative (0) and IRS 3–12 = positive (1))				IRS = Immune Reactive Score, Bon = Bonferroni P1 = Parameter 1 P2 = Parameter 2	
DKK1	HPV	0.0012	0.0114		
CD15	HPV	0.0291	0.2907		
DKK1	CD15	0.3411	1.0000		
p63	CD15	0.3255	1.0000		
p63	CD147	0.1489	1.0000		
CD147	HPV	1.0000	1.0000		
p63	DKK1	1.0000	1.0000		
p63	HPV	1.0000	1.0000		
CD147	DKK1	0.6598	1.0000		
CD147	CD15	0.4103	1.0000		

P1	P2	Support	Lift	Confidence (P1:P2)	Confidence (P2:P1)
Association analyses (based on binarized IRS-based calculations (IRS 0–2 = negative (0) and IRS 3–12 = positive (1))					
DKK1	HPV	0.3594	1.3334	0.5000	0.9584
CD15	HPV	0.3226	1.2602	0.4879	0.8334
DKK1	CD15	0.5358	1.0527	0.7143	0.7895
p63	CD15	0.6291	1.0347	0.6843	0.9513
p63	CD147	0.8000	1.0298	0.8800	0.9362
CD147	HPV	0.3385	1.0000	0.4000	0.8462
p63	DKK1	0.6780	0.9933	0.7408	0.9091
p63	HPV	0.3381	0.9931	0.3637	0.9231
CD147	DKK1	0.6123	0.9691	0.7318	0.8109
CD147	CD15	0.5687	0.9604	0.6591	0.8286

*IRS* immune reactive score, *Bon* Bonferroni, *P1* parameter 1, *P2* parameter 2.

Next, IRS were categorized as previously^11–13^ (IRS 0–2 were defined as 0 for negative, and IRS 3–12 were defined as 1 for positive) and based on these binarized IRS, we used the Fisher's exact test for all biomarker pairs (Table 1). Before adjustment for multiple comparisons, significance was achieved for HPV + CD15 and HPV + DKK1 and after adjustment using the Bonferroni method for HPV + DKK1, suggesting a dependency.

Association analyses discover relationships and patterns between variables in data sets using the three concepts of Support, Confidence, and Lift and were conducted using the binarized IRS (Table 1). Support defines the fraction of samples, for which both considered markers are positive. The highest Support was retrieved for CD147 + p63, followed by p63 + DKK1 and p63 + CD15, suggesting a higher frequency for double-positive specimens. Confidence is a measure used to quantify the likelihood of the co-occurrence of two biomarkers. Specifically, it calculates the percentage of samples where the first biomarker is positive, and then further calculates the percentage, from these selected samples, for which the second biomarker is positive as well. This provides insight into the strength of the association between the two biomarkers within a subset of samples defined by the presence of the first biomarker. Confidence is asymmetric and tests relationships in both directions. The high Confidence for HPV + p63 but low for p63 + HPV indicated a high frequency of p63 + specimens in case of an HPV + status (Table 1). The findings imply that HPV triggers p63 expression, as corroborated by previous outcomes involving HPV + PeCa cell lines (11). However, in this scenario, the relationship between p63 and HPV appears to be one-way, with p63 dependent on HPV. Notably, there exists an HPV-independent inducer of p63, as evidenced by the presence of HPV- p63 + specimens.

Similarly, the Confidence for HPV + DKK1, HPV + CD147, and HPV + CD15 was above 80%, while the reciprocal analyses retrieved low Confidence. The analyses revealed a high frequency of HPV + specimens that are positive for one or two additional parameters (p63, CD15, CD147, DKK1), pointing to HPV as critical moving force in the carcinogenesis underlying molecular pathways. The low Confidence for the reciprocal pairwise comparisons may indicate the presence of independent inducers of all four parameters. The high Confidence for the parameter pairs CD15 + p63, CD15 + CD147, and all parameters to p63 suggested additional levels of dependency.

The concept of Lift (Table 1) is a ratio of the observed by the expected Support if both markers were assumed independent. Lift values greater than one indicate a positive association and were retrieved for the pairs HPV + DKK1 and HPV + CD15 suggesting an association of both CD15 and DKK1 with HPV. Thus, the three rule-based association measures suggested a notable but not mandatory dependency between the tested parameters depending on the HPV status.

**Fig. 1:**
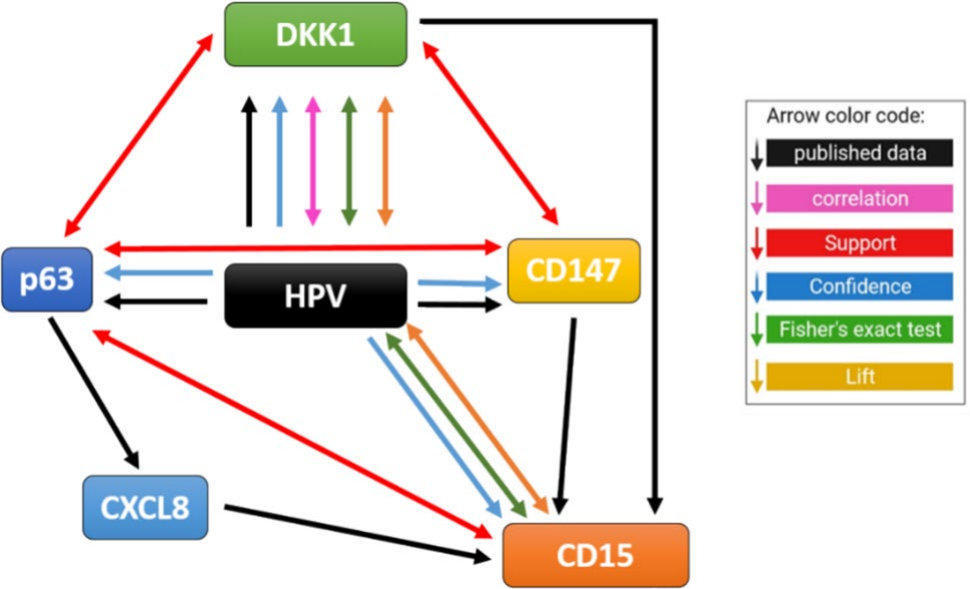
Schematic illustration of relationships between individual biomarkers.

Incorporating all findings^11–13,24,25^ points to HPV oncoproteins as potential key drivers that can induce p63, CD147, CD15, and DKK1 (Fig. 1). Moreover, p63 and CD147 emerge as secondary drivers, acting independently or relying on HPV induction, for the recruitment of CD15 + cells. Remarkably, DKK1 demonstrates a particularly strong connection with HPV. Notably, the correlations among CD147, DKK1, and p63 are intertwined due to their shared dependency on HPV, casting doubt on their potential as distinct biomarkers or suggesting a level of interchangeability. High-risk HPV oncoproteins, particularly with CD147 and TAN, exhibit a noteworthy capacity to drive metastatic cancers individually, hinting at a possible synergistic effect if combined. However, a comprehensive survival analysis is essential to elucidate the most promising individual biomarker or a synergistic combination.

### The impact of HPV-p63-CD15 status on prognosis of PeCa patients

**Fig. 2:**
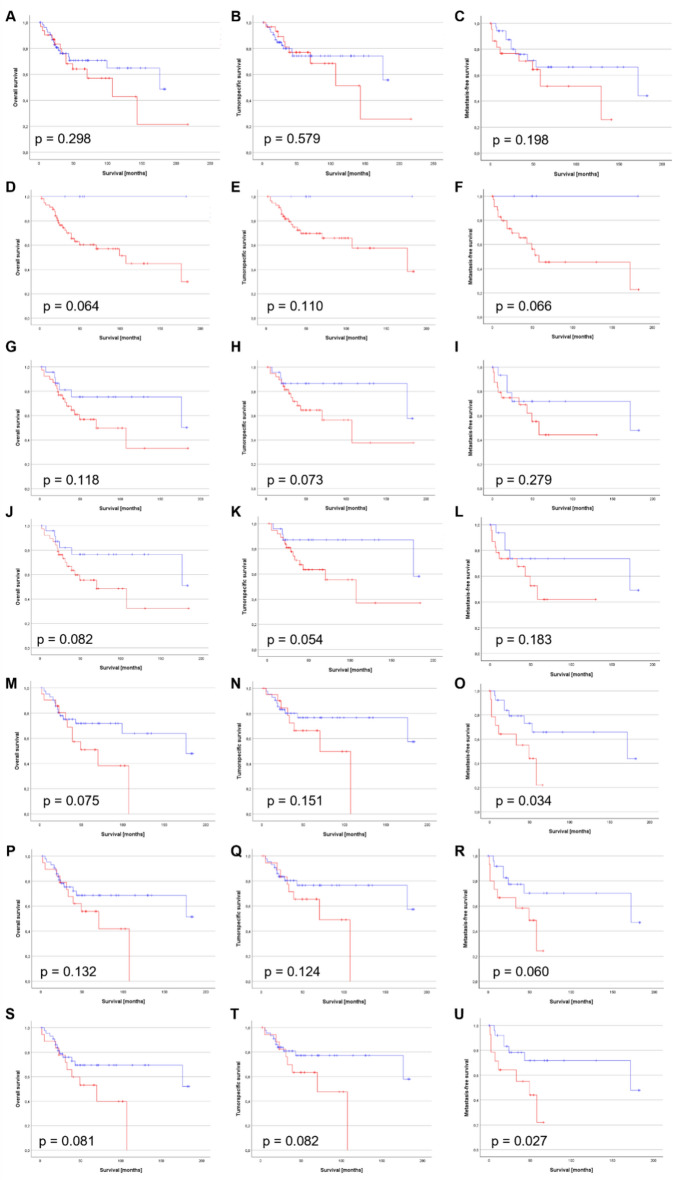
Survival estimation by Kaplan–Meier for the HPV-p63-CD15 status. Kaplan–Meier survival statistics were calculated using the Log-Rank test for HPV + (**A**–**C**), p63 + (**D**–**F**), CD15 + (**G**–**I**), p63 + CD15 + (**J**–**L**), HPV + p63 + (**M**–**O**), HPV + CD15 + (**P**–**R**) and HPV + p63 + CD15 + (**S**–**U**) PeCa specimens for OS (**A**,**D**,**G**,**J**,**M**,**P**,**S**), TSS (**B**,**E**,**H**,**K**,**N**,**Q**,**T**) and MFS (**C**,**F**,**I**,**L**,**O**,**R**,**U**). Red lines = positive, blue lines = negative.

PeCa patients with elevated levels of infiltrating neutrophils are at higher risk for invasive growth and metastatic cancers^11^. The recently described infiltration of CD15 + immune cells into HPV + and p63 + PeCa specimens was prominent in the tumor center in contrast to the invasion front suggesting that the tumor immune biology at the center may impact clinical outcome and survival^11,17^. We conducted Kaplan–Meier estimates to test the capacity of the previously identified markers, HPV, p63, CD15, to affect survival. The HPV status itself had no significant impact on survival (Fig. 2A–C). P63 (Fig. 2D–F), CD15 (Fig. 2G–I), the combination of both (Fig. 2J–L), and of each with the HPV + status (HPV + p63, Fig. 2M–O; HPV + CD15, Fig. 2P–R) affected the survival of PeCa patients. Notably, PeCa specimens with an HPV + p63 status had a significantly reduced MFS with a trend towards survival disadvantage regarding OS and TSS. The combination of all three, HPV + p63 + CD15, significantly reduced the MFS and impacted OS and TSS as calculated by Kaplan–Meier estimates (Fig. 2S–U). Previous results demonstrated that patients with a triple positive status (HPV + p63 + CD15) are at high risk of dedifferentiated, invasive growing, and metastasizing cancers^11^. The survival estimates presented here provided further hints that the underlying carcinogenesis fueling molecular feedback loop may mark patients at high risk for negative survival prognosis.

### The impact of DKK1 on the survival of PeCa patients

**Fig. 3:**
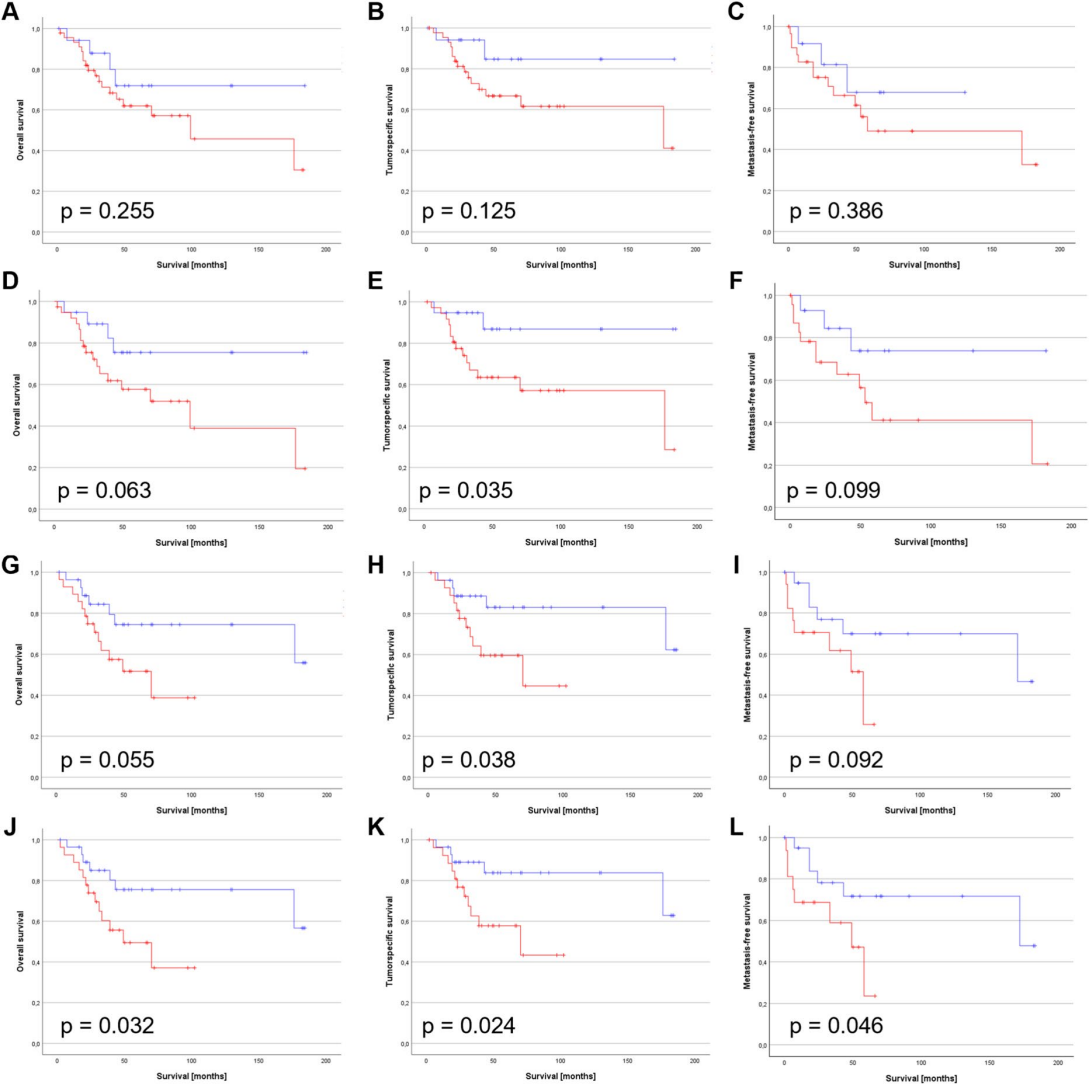
Survival estimation by Kaplan–Meier of the DKK1-p63-CD15 status. Kaplan–Meier survival statistics were calculated using the Log-Rank test for DKK1 + (**A**–**C**), DKK1 + p63 + (**D**–**F**), DKK1 + CD15 + (**G**–**I**), DKK1 + p63 + CD15 + (**J**–**L**) PeCa specimens for OS (**A**,**D**,**G**), TSS (**B**,**E**,**H**) and MFS (**C**,**F**,**I**). Red lines = positive, blue lines = negative.

While neutrophils are important players in cancer progression and negatively predict clinical prognosis and survival in HPV-associated entities, the local TME plays a critical role in reshaping cancer cell and neutrophil plasticity and function^1–3^. DKK1 was suggested to keep PeCa cells in a stemness-like hybrid state between dormancy and metastatic outbreak^12,26^ and as a driver for invasive growing and metastasizing PeCa with potential as a putative biomarker^12^. Subsequent Kaplan–Meier survival estimates based on the binarized IRS suggested that DKK1 alone might have to some extend potential to predict the survival of patients (Fig. 3A–C). The combination of DKK1 either with p63 (Fig. 3D–F) or CD15 (Fig. 3G–I) and with both p63 and CD15 (Fig. 3J–L) significantly affected the OS, TSS, and MFS of PeCa patients. Supplementation of HPV displayed no benefit in predicting survival (Suppl. Fig. S1).

In summary, these data suggest that patients with a tumor biology characterized by DKK1, p63 and CD15, have a reduced survival prognosis due to more aggressive growing cancers, as previously suggested^11,12^. DKK1 has been shown to increase the burden of NDSC^27^. While the p63-based induction of CXCL8 can foster the infiltration of neutrophils into the TME^11^, DKK1 may promote their polarization into NDSC that in turn further fuel disease progression by facilitating invasion and metastatic outbreaks.

### A CD147 + CD15 + driven TME is associated with reduced survival of PeCa patients

CD147 was initially identified by a human cytokine array as differentially expressed in conditioned media of HPV + PeCa cell lines^11^. Subsequent IHC staining of a TMA with tumor center bunches of PeCa specimens pointed to CD147 as a putative marker for invasive and metastasizing PeCa, in particular in case of an HPV + status^13^. CD147 expression is enhanced by active HPV oncoproteins and is associated with poor prognoses in other HPV-related entities^13,28–32^ underlining its potency to predict survival.

**Fig. 4:**
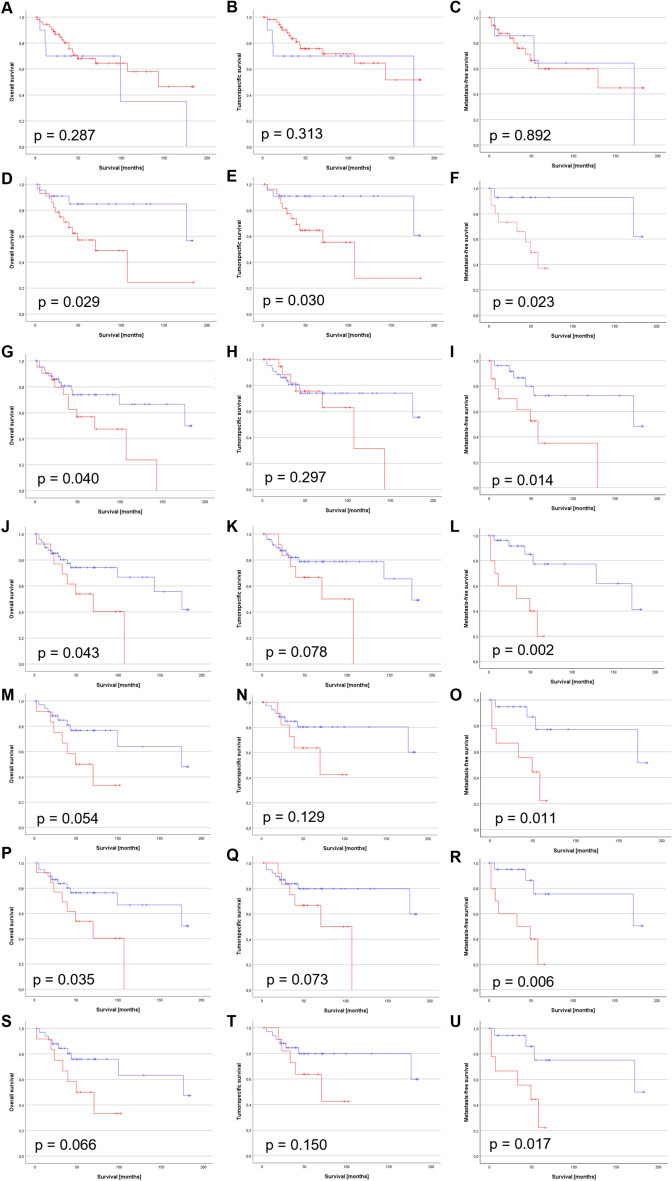
Survival estimation by Kaplan–Meier of the HPV-CD147-CD15 status. Kaplan–Meier survival estimates were calculated using the Log-Rank test for CD147 + (**A**–**C**), CD147 + CD15 + (**D**–**F**), HPV + CD147 + (**G**–**I**) and HPV + CD147 + CD15 + (**J**–**L**), HPV + CD147 + CD15 + DKK1 + (**M**–**O**), HPV + CD147 + CD15 + p63 + (**P**–**R**) and HPV + CD147 + CD15 + p63 + DKK1 + (**S**–**U**) PeCa specimens for OS (**A**,**D**,**G**,**J**,**M**,**P**,**S**), TSS (**B**,**E**,**H**,**K**,**N**,**Q**,**T**) and MFS (**C**,**F**,**I**,**L**,**O**,**R**,**U**). Red lines = positive, blue lines = negative.

While CD147 alone had no impact on the survival of patients (Fig. 4A–C), Kaplan–Meier survival estimates based on the previously published TMA staining data^13^ underlined the potential of the CD147 + CD15 (Fig. 4D–F) and HPV + CD147 (Fig. 4G–I) status as both negatively predicted OS and MFS of PeCa patients, with the CD147 + CD15 status impacting TSS as well. CD147 mediates chemotaxis of neutrophils and previous data implicated a central role of a CD147-neutrophil axis in the (HPV-driven) penile carcinogenesis^13^. The data landscape so far suggests a different and significantly more adverse tumor biology if CD147 is induced by viral oncoproteins and may amplify TAN-fueled carcinogenesis^13^. Subsequent Kaplan–Meier survival estimates of the combined parameters (HPV + CD147 + CD15) indicated a significant impact of this marker profile on the OS and MFS of PeCa patients (Fig. 4J–L). Next, we evaluated if DKK1 (Fig. 4M–O) and p63 (Fig. 4P–R) alone or combined (Fig. 4S–U) may affect these survival estimates. All the variants predicted MFS while the HPV + CD147 + CD15 + p63 status significantly reduced OS as well. Notably, the Kaplan Meier survival estimates underlined the potential of the CD147 + CD15 status to predict the survival of PeCa patients with a potential of further markers to improve prediction of MFS (HPV, HPV + p63, HPV + DKK1, HPV + p63 + DKK1) or predicting OS (HPV, HPV + p63) too. After adjustment of p values for multiple comparison, the HPV + CD147 + CD15 status remained the most potent profile affecting MFS (Suppl. Table S1).

### Cox regression analyses underlined the potency of the HPV-CD147-CD15 biomarker profile predicting survival of patients

Next, Cox regression analyses were conducted to further estimate the potential and to estimate the Hazard ratios (HR) of the individual biomarkers and combinations as predictors of survival.

The results of the univariable Cox regression analyses (Table 2) suggested an impact of the transcription factor p63 on survival. CD15 alone displayed a non-significant trend for reduced OS and TSS (Table 2). The combination of p63 and CD15 showed a non-significant tendency for reduced OS and TSS (Table 2). The combination of HPV + p63 resulted in a non-significant trend regarding OS and a significant impact on MFS. HPV + CD15 was in tendency linked with reduced MFS, while triple-positive patients (HPV + p63 + CD15) had a significantly reduced OS and MFS (Table 2). On the other side, clinical parameters (nodal (N), invasion (I), lymphovascular (L), vascular (V), and perineural (Pn) invasion) displayed a notable potency to predict survival (Suppl. Figs. S2, S3). In multi-variable analyses including clinical invasion parameters (L, V), patients with HPV + p63 + CD15 positive PeCa had a significantly reduced OS (Table 2). Inclusion of the nodal status revealed similar results regarding the potency of the triple-positive HPV + p63 + CD15 status to predict a reduced MFS (Table 2).

**Table 2:** Cox proportional hazard regression analysis.

Parameter	Univariable Cox regression					
	MFS		TSS		OS	
	HR (95% CI)	p	HR (95% CI)	p	HR (95% CI)	p
Individual parameters and combinations						
HPV	1.820 (0.719–4.604)	0.206	1.272 (0.541–2.992)	0.581	1.474 (0.706–3.077)	0.302
p63	27.863 (0.095–8170.239)	0.251	25.126 (0.051–12,273.892)	0.307	25.076 (0.120–5259.497)	0.238
CD15	1.871 (0.586–5.980)	0.290	2.731 (0.872–8.554)	0.085	2.101. (0.81–5.445)	0.127
p63 + CD15	2.150 (0.673–6.686)	0.197	2.924 (0.937–9.121)	0.065	2.270 (0.878–5.869)	0.091
HPV + p63	2.854 (1.029–7.914)	**0.044**	1.966 (0.761–5.235)	0.160	2.085 (0.911 + 4.773)	0.082
HPV + CD15	2.663 (0.917–7.730)	0.072	2.092 (0.798–5.482)	0.133	1.895 (0.812–4.420)	0.139
HPV + p63 + CD15	3.483 (1.074–9.023)	**0.037**	2.288 (0.875–5.986)	0.092	2.764 (0.895–4.856)	**0.040**
DKK1	1.733 (0.488–6.145)	0.395	3.001 (0.686–13.133)	0.145	1.858 (0.628–5.497)	0.263
DKK1 + CD15	2.541 (0.82–7.868)	0.106	3.180 (1.005–10.063)	**0.049**	2.507 (0.948–6.633)	0.064
DKK1 + p63	2.761 (0.778–9.800)	0.116	4.135 (0.977–19.046)	0.054	2.702 (0.906–8.061)	0.075
HPV + DKK1	1.537 (0.554–4.260)	0.409	1.445 (0.537–3.891)	0.466	1.457 (0.609–3.486)	0.397
HPV + DKK1 + CD15	2.257 (0.753–6.769)	0.146	1.809 (0.653–5.007)	0.254	1.830 (0.741–4.516)	0.190
DKK1 + p63 + CD15	2.974 (0.961–9.199)	0.059	3.483 (1.102–11.010)	**0.034**	2.764 (1.045–7.312)	**0.040**
HPV + DKK1 + p63 + CD15	2.661 (0.889–7.963)	0.080	2.007 (0.726–5.550)	0.179	2.042 (0.828–5.036)	0.121
CD147	0.915 (0.253–3.309)	0.893	0.567 (0.186–1.734)	0.320	0.587 (0.217–1.587)	0.294
CD147 + CD15	7.672 (0.959–61.368)	0.055	3.893 (1.050–14.433)	**0.042**	3.349 (1.064–10.540)	**0.039**
HPV + CD147	3.659 (1.212–11.480)	**0.024**	1.668 (0.631–4.412)	0.302	2.350 (1.013–5.455)	**0.047**
HPV + CD147 + CD15	5.633 (1.643–19.305)	**0.006**	2.471 (0.874–6.986)	0.088	2.453 (0.997–6.032)	0.051
HPV + CD147 + CD15 + DKK1	5.051 (1.258–20.278)	**0.022**	2.435 (0.742–7.989)	0.142	2.621 (0.946–7.264)	0.064
HPV + CD147 + CD15 + p63	5.457 (1.408–21.151)	**0.014**	2.628 (0.877–7.877)	0.085	2.693 (1.032–7.022)	**0.043**
HPV + CD147 + CD15 + p63 + DKK1	4.643 (1.158–18.621)	**0.030**	2.333 (0.711–7.651)	0.162	2.515 (0.907–6.973)	0.076
Clinical parameters						
Nodal status*	2.528 (1.315–4.857)	**0.005**	3.198 (1.738–5.885)	** < 0.001**	2.607 (1.538–4.421)	** < 0.001**
Invasion status**	1.474 (0.715–3.038)	0.293	2.072 (0.989–4.339)	0.053	1.792 (0.978–3.282)	0.059
Vascular invasion (V)	1.820 (0.832–3.985)	0.134	2.874 (1.489–5.547)	**0.002**	2.420 (1.336–4.383)	**0.004**
Perineural invasion (Pn)	3.722 (1.332–10.398)	**0.012**	7.058 (2.976–16.738)	** < 0.001**	5.197 (2.277–11.861)	** < 0.001**
Lymphovascular invasion (L)	2.942 (1.263–6.851)	**0.012**	3.099 (1.460–6.578)	**0.003**	3.007 (1.529–5.912)	**0.001**

	Multi-variable Cox regression					
	MFS		TSS		OS	
	HR (95% CI)	p	HR (95% CI)	p	HR (95% CI)	p
Parameter compared to (N, V, L)						
HPV + p63 + CD15	4.162 (1.245–13.909)	n.d	n.d	n.d	n.d	n.d
HPV + CD147 + CD15	n.d	n.d	3.188 (1.020–9.965)	**0.046**	3.140 (1.171–8.420)	**0.023**
HPV + CD147 + CD15 + p63	7.341 (1.522–35.405)	**0.013**	3.461 (1.047–11.434)	**0.042**	3.229 (1.164–8.962)	**0.024**
HPV + CD147 + CD15 + DKK1 + p63	6.153 (1.239–30.59)	**0.026**	n.d	n.d	3.112 (1.040–9.313)	**0.042**
Parameter compared to (N, V, L, HPV + CD147 + CD15 + DKK1)						
HPV + p63 + CD15	6.796 (1.366–33.798)	**0.019**	n.d	n.d	3.252 (1.089–9.742)	**0.035**
Parameter compared to (L, V)						
HPV + p63 + CD15	4.162 (1.245–13.909)	**0.021**	n.d	n.d	3.300 (1.168–9.492)	**0.024**
Parameter compared to (L)						
DKK1 + p63 + CD15	n.d	n.d	3.311 (1.048–10.456)	0.041	2.628 (0.994–6.945)	0.051
Parameter compared to (N, V, Pn, L)						
HPV + CD147 + CD15	6.292 (1.217–32.520)	**0.028**	n.d	n.d	n.d	n.d
CD147 + CD15	54.081 (0.175–16,704.581)	0.172	10.903 (1.376–86.383)	**0.024**	6.155 (1.361–27.829	**0.018**

*L* lymphovascular invasion, *V* vascular invasion, *Pn* perineural invasion.

*Nodal status: non-metastases: pN0 + cN0, metastases = pN1-4.

**Invasion status: non-invasive = pTis-pT1a, invasive = pT1b-pT4; n.d. = not determined.

Bold entries: p ≤ 0.05.

Univariable testing revealed a significant survival disadvantage of patients with DKK1 + CD15 positive PeCa regarding TSS and a similar trend for OS, while DKK1 alone displayed an increased HR for TSS (Table 2). The DKK1 + p63 + CD15 status was significantly associated with reduced OS, TSS, and a tendency for MFS (Table 2). In multi-variable analyses, the DKK1 + p63 + CD15 status patients displayed a notable trend for an OS and TSS (Table 2) disadvantage if compared to lymphovascular invasion (L) but not to any other clinical parameter (N, I, V, Pn) or all clinical parameters combined.

Univariable Cox regression analyses revealed a significant prognostic value of the HPV + CD147 status for OS and MFS (Table 2), and a non-significant trend for OS and for TSS (Table 2). There was a significant result for MFS of the HPV + CD147 + CD15 status (Table 2). Combination with p63 or DKK1 or both resulted in a significantly reduced MFS and OS for patients with HPV + CD147 + CD15 + p63 cancers (Table 2). Notably, the CD147 + CD15 status was potent in predicting a significantly reduced OS, TSS, and MFS in Kaplan Meier survival estimates (Fig. 3D–F) and for OS and TSS in respective Cox regression analyses with a high HR for MFS (OS: HR: 3.349 (1.064–10.540) p = 0.039; TSS: HR: 3.893 (1.050–14.433), p = 0.042; MFS: HR: 7.672 (0.959–61.368), p = 0.055). Multi-variable Cox regression analyses revealed a significant prognostic value of the combination of HPV, CD15, and CD147 compared to clinical parameters (N, I, L, V, Pn) for MFS (Table 2), compared to clinical parameters (N, I, L, V) for OS and TSS (Table 2). The addition of p63 (HPV + CD15 + CD147 + p63) resulted in a significantly reduced OS, TSS and MFS (Table 2) if compared to clinical parameters (N, I, L, V). Inclusion of DKK1 instead (HPV + CD15 + CD147 + DKK1) revealed a significant impact on MFS and OS (Table 2) for the HPV + CD147 + CD15 status compared to clinical parameters (N, I, L, V). Patients with quintuple positive cancers (HPV + CD147 + CD15 + p63 + DKK1) displayed a significantly reduced OS and MFS (Table 2) compared to clinical parameters (N, I, L, V).

Notably, multi-variable Cox regression analyses revealed a significant potency of the CD147 + CD15 status to predict OS and TSS and a high likelihood for reduced MFS, although not significant (Table 2), compared to the HPV status and clinical parameters (N, I, L, V).

In summary, PeCa specimens positive for HPV, CD15, and CD147 reflected patients with notably reduced MFS (after Bonferroni correction for multiple comparison (Suppl. Table S1)), with a potential of the CD147 + CD15 combination without HPV in predicting survival (OS, TSS), while the benefit of p63 and DKK1, as additional biomarkers remained vague requiring further testing.

### Association analyses to evaluate the potency of biomarkers and their combinations

The Confidence measure (based on its if–then structure) allows the evaluation of the benefit of adding additional biomarkers to an existing biomarker combination. A Confidence of 1 indicates that all cases positive for one biomarker combination are positive for the tested additional biomarker and consequently adding the additional biomarker does not provide any further information nor benefit. The Confidence for p63 compared to the combinations CD15 + CD147, CD15 + CD147 + DKK1, HPV + CD15 + CD147 + DKK1, and HPV + CD15 + CD147, and for DKK1 compared to the profiles HPV + CD15 + CD147, HPV + CD15, HPV + p63 + CD15, and HPV + CD147 + p63 + CD15 was 1. The lower Confidence of each single biomarker combination to HPV suggested a potential to add the HPV + status. Thus, based on our data, p63 and DKK1 cannot improve survival prognosis beyond the combination of HPV + CD15 + CD147 (Suppl. Table S2), as the information is already contained in the triple combination reflected by the Confidence results pointing to no further prognostic value of adding them.

After all, the CD147 + CD15 and HPV + CD147 + CD15 status could display separate tumor pathogeneses, while the HPV + CD15 + CD147 status remained the most potent to predict survival (MFS) of PeCa patients.

## Discussion

PeCa is a rare malignant disease with rising incidence rates while research material is limited. This study examined biomarkers (p63, CD15, DKK1, CD147) for their predictive value in PeCa patient survival, using scarce resources, as TMA with clinical data. These parameters were linked with advancing disease and reduced survival, with active expression of functional viral oncoproteins as the main regulator and CD147 and CD15 as synergistic parameters. This underscores the importance of including biomarkers beyond the HPV status that reflect the actual tumor biology to improve prognosis^33^.

The candidate biomarkers and profiles evaluated^11–13^ emphasize the importance of a neutrophil-recruiting TME in PeCa progression. PeCa cells release CXCL8, DKK1, and CD147, leading to reprogrammed fibroblasts and TAN promoting cancer progression and metastasis^2,12,34–36^. The formation of (distant organ) metastases is the cause of most cancer-related deaths underscoring the urgent need to identify drivers. CD147 and CD15 (and HPV +) displayed potential to predict the MFS suggesting complementary amplification loops and displaying promising candidates. Targeting the immunosuppressive myeloid cell compartment could display a promising strategy.

The data presented here supported the prominent cancer-promoting potential of TAN in the tumor center of PeCa. Previous reports identified CD147 + or DKK1 + PeCa specimens especially with high IRS at the tumor center^12,13^ and suppressive neutrophils in the tumor core of head and neck cancer^17^ as strongly associated with metastases, advanced-stage disease, inferior OS and disease-free survival^2,22,23,27–31,37,38^ and therapeutic resistance^1,2,17,32^. TAN phenotypes are encountered differently in the tumor center and invasion front with differentially polarized neutrophils that support tumor growth and invasion at a given time^2,22^. The spatial context and interplay of myeloid and lymphoid immune cells at the tumor margin compared to the tumor center could be critical to understand which type of compromised immune cell supports penile carcinogenesis at which step^39^.

PeCa present as histological and pathogenetic different HPV-related and non-related subtypes^10–13,40^. However, not all HPV-related subtypes are HPV + and a part of the usual type squamous cell cancers (SCC) are HPV + , requiring a HPV status definition by the expression of functional viral oncoproteins^10–13^. An HPV + status has been associated with a more favorable disease-specific survival in retrospective studies^41–44^. Others reported similar Kaplan–Meier and Cox regression survival estimates for HPV + and HPV- PeCa^45^. The detection of HPV DNA together with p16INK4A defined biologically and clinically distinct types of oropharyngeal SCC that affect therapeutic response^46,47^. Our findings suggest that the HPV status and its definition are clinically relevant in case of PeCa, like in other HPV-related conditions^46,47^, and point to biologically and clinically distinct entities also among the HPV + group and the HPV + vs. HPV- groups^48^. Survival estimates in HPV- and HPV + groups indicated a survival disadvantage if positive for p63 and opposing prognoses regarding CD147 (Suppl. Figs. S3–S8, Suppl. Table S3). Finally, the combinations (DKK1 + p63 + CD15 + , CD147 + CD15) could display promising biomarker candidates independent of the HPV status. Standardized protocols regarding study design, sample handling and definition of HPV status would be of great help in harmonizing data and survival estimates.

The association analyses revealed a partial and notable but not binding inter-dependency pointing to chain reactions, where one biomarker can induce others. Computational modelling can delineate the impact of cancer-immune dynamics on patient survival. With a strong link between the transition of tumors from immune control to immune evasion as a (time-) critical parameter affecting survival and therapeutic response^49^. The combination of immune- and tumor-related biomarkers may help tailoring immunotherapeutic approaches, as composite scores had a 10–20-fold higher potency to predict progressing disease^50^. Multi-parameter signatures combined with robust computational methods may outperform validated clinical molecular tests in predicting patient outcomes^51,52^. Conversely, these multi-parameter scores may help to identify patients with a potential survival advantage or disadvantage based on the absence or presence of biomarkers such as those identified here.

The study's limitations include its retrospective nature, small sample size, and numerous tests, which affect statistical robustness. The high number of tests increases the risk of non-significant results despite adjustments for multiple comparisons. Presenting both non-adjusted and adjusted p-values helps to balance statistical requirements and sample size. The required sample size for robust statistics is much higher than in this study, though the sample size for CD147 + CD15 and HPV + CD147 + CD15 profiles was sufficient (Suppl. Table S4). However, the small sample size limits the ability to robustly estimate survival within specific HPV-related and non-related subtypes. Collaborating with multiple centers is crucial for collecting enough samples to achieve the necessary statistical power.

In summary, neutrophil infiltration and a CD147-driven TME are hallmarks of cancer progression, including HPV-driven penile carcinogenesis. The HPV + CD147 + CD15 biomarker profile is a promising candidate for further study with larger sample sizes. The combined biomarkers significantly impact survival, suggesting a synergistic effect that enhances predictive potential. Future research on the TME and immune cell subsets could lead to more precise and personalized treatment strategies.

## Methods

### Ethical statement, cohort, and study design

The local Ethics Committee of the Saarland (Ärztekammer des Saarlandes, Saarbrücken, Germany) in accordance with the Declaration of Helsinki approved experiments with human material used in this study and written informed consent by study participants. The TMA cohort consists of patients derived from Russia and Germany between 1992 and 2015. Data on clinical outcome and HPV status were published previously^10,11^. Briefly, DNA was isolated from FFPE tissue sections by QIAamp DNA FFPE Tissue Kit (Qiagen, Hilden, Germany) following manufacturers protocol and the HPV PCR was conducted using the GP5 + /6 + primers as described previously^10,11^. HPV status was further determined by p16INK4A immunohistochemistry, a surrogate marker for HPV oncoprotein driven transformation, using a published protocol^10,11^. HPV status was considered as positive in case of positive PCR and IHC. Sections of all cases were reviewed by two experienced uropathologists and histological subtypes as well as tumor grade were defined according to the 2016 WHO classification and the 8th editions of TNM classification of malignant tumors. Basic cohort description and patient/disease characteristics are listed in Suppl. Table S5. The respective tissue was punched for the individual TMA reflecting tumor center in duplicates. Results of IHC on respective biomarkers have been published previously^11–13^. While DKK1, CD147, p16INK4A, S100A8 and S100A9 were stained as single staining, CD15 and p16 were co-stained. Representative positively and negatively stained images of the individual manual staining were collected in supplementary Fig. S9.

### Statistical analyses

Statistical data on nodal status along other clinic-pathological parameters have been published previously^10^. Statistical survival analyses were performed using SPSS (version 29.0.0.0 (241)). Kaplan–Meier estimator and Log-Rank test (Mantel-Cox) were per-formed for survival (overall survival (OS), tumor-specific survival (TSS), metastases-free survival (MFS)) analyses. Kaplan–Meier plots were generated using SPSS and respective numbers-at-risk were summarized in Supplementary Table S6. Uni- and multi-variable analyses were performed by Cox proportional hazard analysis. Dependency testing was conducted by calculating the Pearson and Distance correlation, the Hoeffding’s and Fisher’s exact test of independence, and three concepts of association analyses (Support, Confidence, Lift)^53–58^ were used to discover similarities and relationships between variables and their combinations based on the previously published immune reactive scores (IRS)^11–13^ using the R packages “energy”, “Hmisc”, “stats”, and for sample size calculation “powerSurvEpi”^59–62^. Residual graphical and statistical analyses were performed using Graph Pad Prism 9.3.1. (Graph Pad Software, San Diego, USA). Group data are reported as mean ± SEM. Significance was determined by two-way or one-way ANOVA repeated measures test with Tukey’s correction. Significance was accepted when p-values were < 0.05. Multiple comparison-adjusted p-values (p_adj) were calculated using Bonferroni (formula: ${p}_{Bon}= p\times \#tests)$ correction for Kaplan Meier survival estimates and Cox regression analyses for OS, TSS and MFS, respectively^58^.

### Ethics approval and consent to participate

The study was conducted in accordance with the Declaration of Helsinki and approved by the local Ethics Committee of the Saarland (Ärztekammer des Saarlandes, Saarbrücken, Germany) (Permit No. 220/19).

## Supplementary Information

Supplementary Information.

## Data Availability

The datasets used and/or analyzed during the current study are available from the corresponding author on reasonable request.
