# Supplementary material for: The impact of the tumor microenvironment on the survival of penile cancer patients
Source: Sci Rep. 2024 Sep 27;14:22050. doi: 10.1038/s41598-024-70855-z (PMC11436934; doi:10.1038/s41598-024-70855-z)
Supplement: Supplementary file 1 — Supplementary Information. [file 41598_2024_70855_MOESM1_ESM.docx]

Supplementary Materials:

**Supplementary Table 1: Adjustment of p values for multiple comparison**

**Supplementary Fig. 1:** Kaplan-Meier survival estimates of HPV+DKK1+ (A-C), HPV+DKK1+CD15+ (D-F) and HPV+DKK1+p63+CD15+ (G-I) PeCa specimens for OS (A, D, G), TSS (B, E, H) and MFS (C, F, I). Estimates were calculated using Log-Rank test. Red lines = positive, blue lines = negative.

**
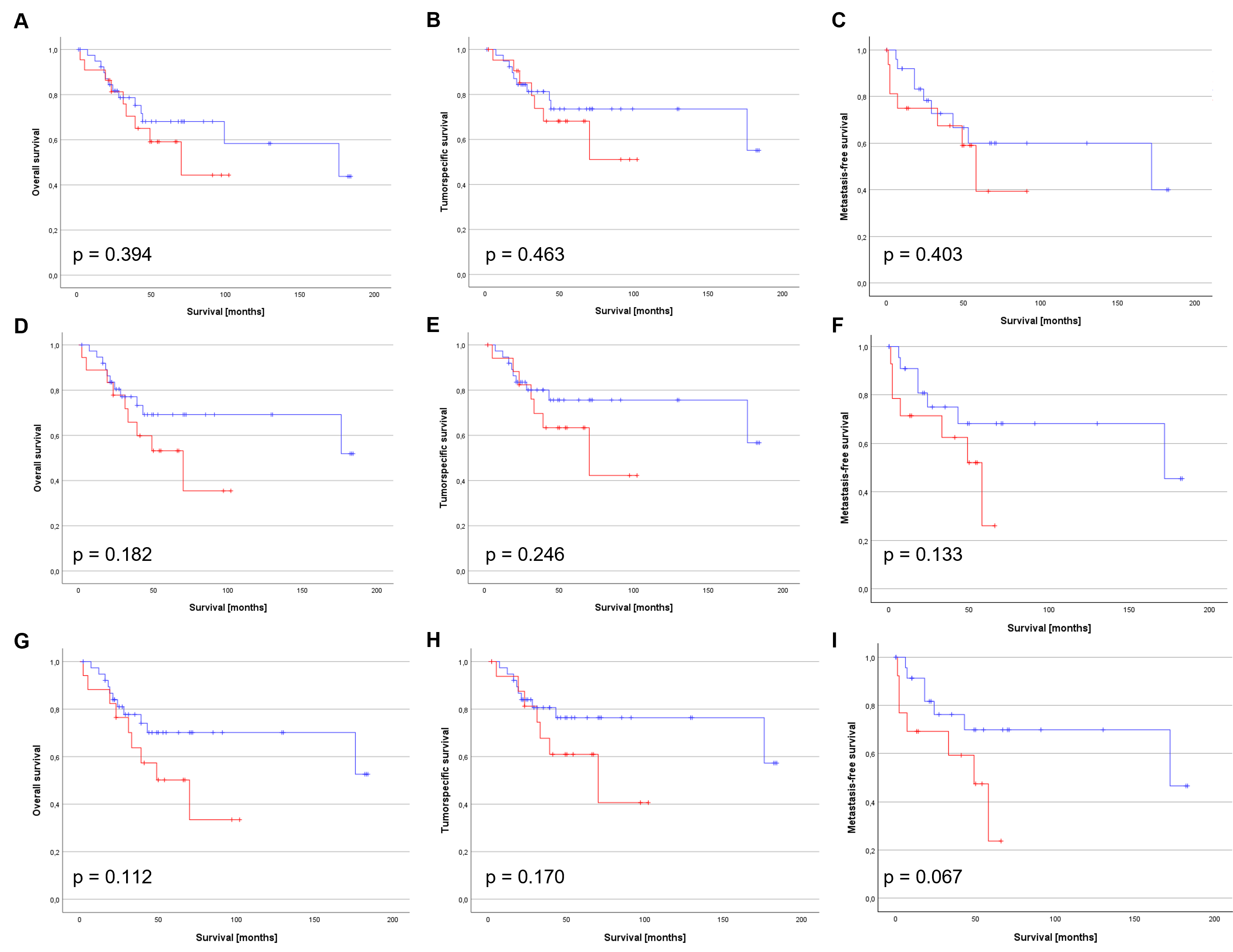
**

**Supplementary Fig. 2:** Kaplan-Meier survival estimates depending on nodal (A-C), invasion status (D-F), lymphovascular invasion (G-I), perineural invasion (J-L) and vascular invasion (M-O) of PeCa specimens for OS (A, D, G, J, M), TSS (B, E, H, K, N) and MFS (C, F, I, L, O). Estimates were calculated using Log-Rank (A-C, G-O) and Breslow (D-F) test. Results using Log-Rank test for D-F were p = 0.005, 0.048 and 0.289 for OS, TSS and MFS, respectively. Nodal status: non-metastases: pN0+cN0, metastases = pN1-4; invasion status: non-invasive = pTis-pT1a, invasive = pT1b-pT4. Red lines = positive, blue lines = negative.

**
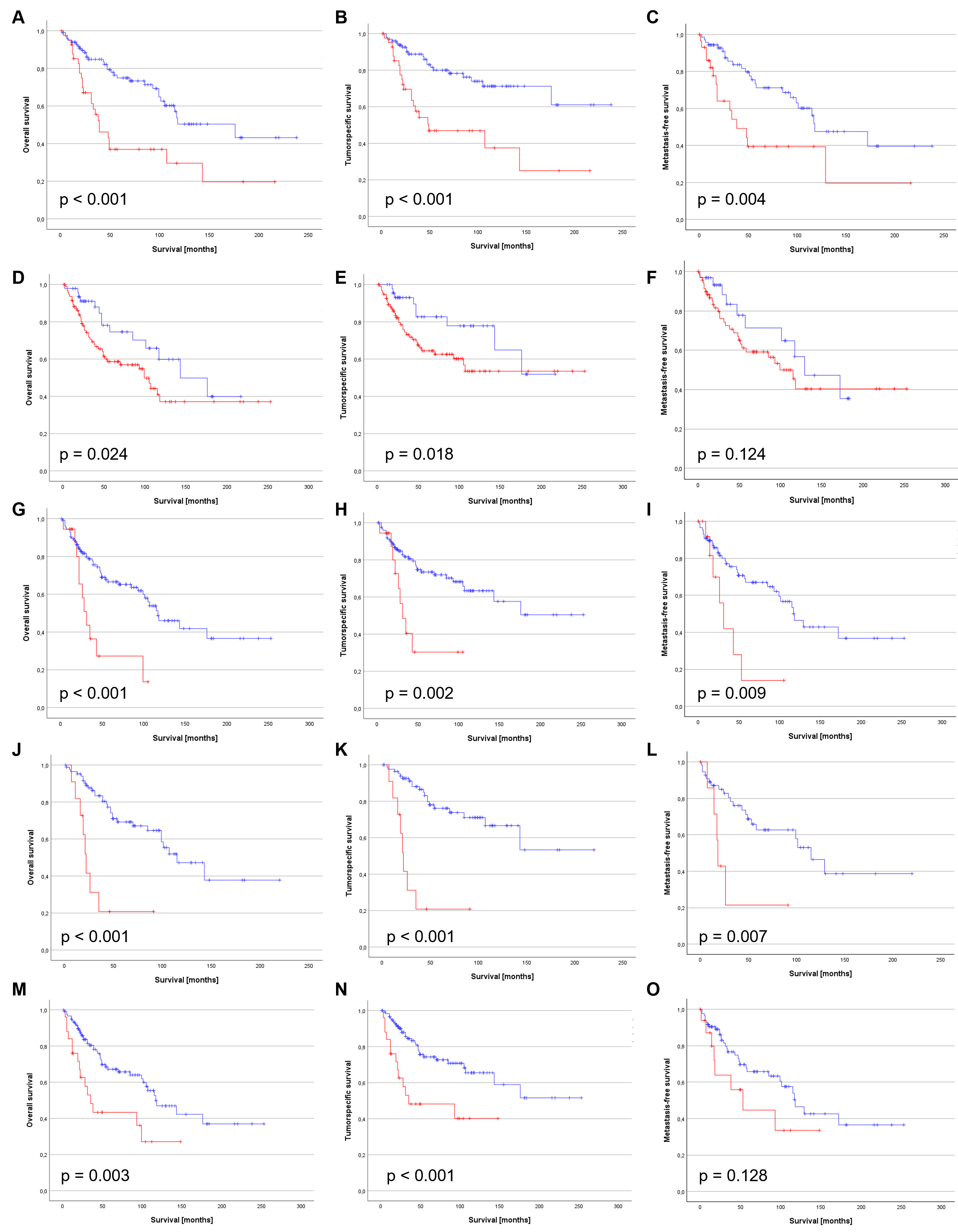
**

**Supplementary Table 2: Inter-dependence testing of parameter combinations with one single parameters:** Confidence and Fishers exact test of independence were calculated. P values were adjusted for multiple comparison using the Bonferroni method.

**Supplementary Figure 3: Kaplan-Meiers survival estimates of HPV- PeCa specimens for p63 and CD15:** Kaplan-Meier survival estimates of p63+ (A-C), CD15+ (D-F) and p63+CD15+ (G-I) HPV- PeCa specimens for OS (A, D, G), TSS (B, E, H) and MFS (C, F, I). Estimates were calculated using Log-rank test. Red lines = positive, blue lines = negative.


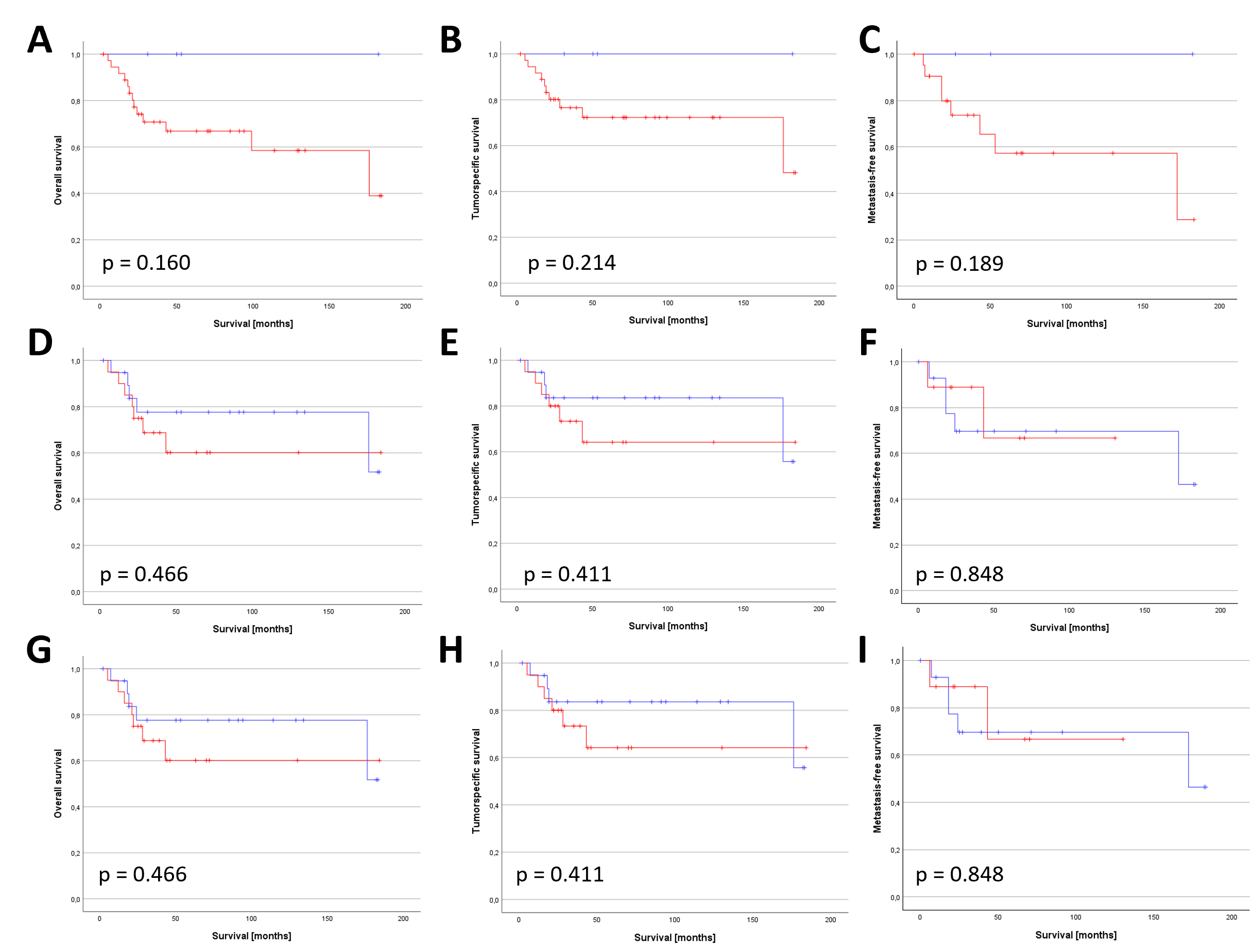


**Supplementary Figure 4: Kaplan-Meiers survival estimates of HPV+ PeCa specimens for p63 and CD15:** Kaplan-Meier survival estimates of p63+ (A-C), CD15+ (D-F) and p63+CD15+ (G-I) HPV+ PeCa specimens for OS (A, D, G), TSS (B, E, H) and MFS (C, F, I). Estimates were calculated using Log-rank test. Red lines = positive, blue lines = negative.


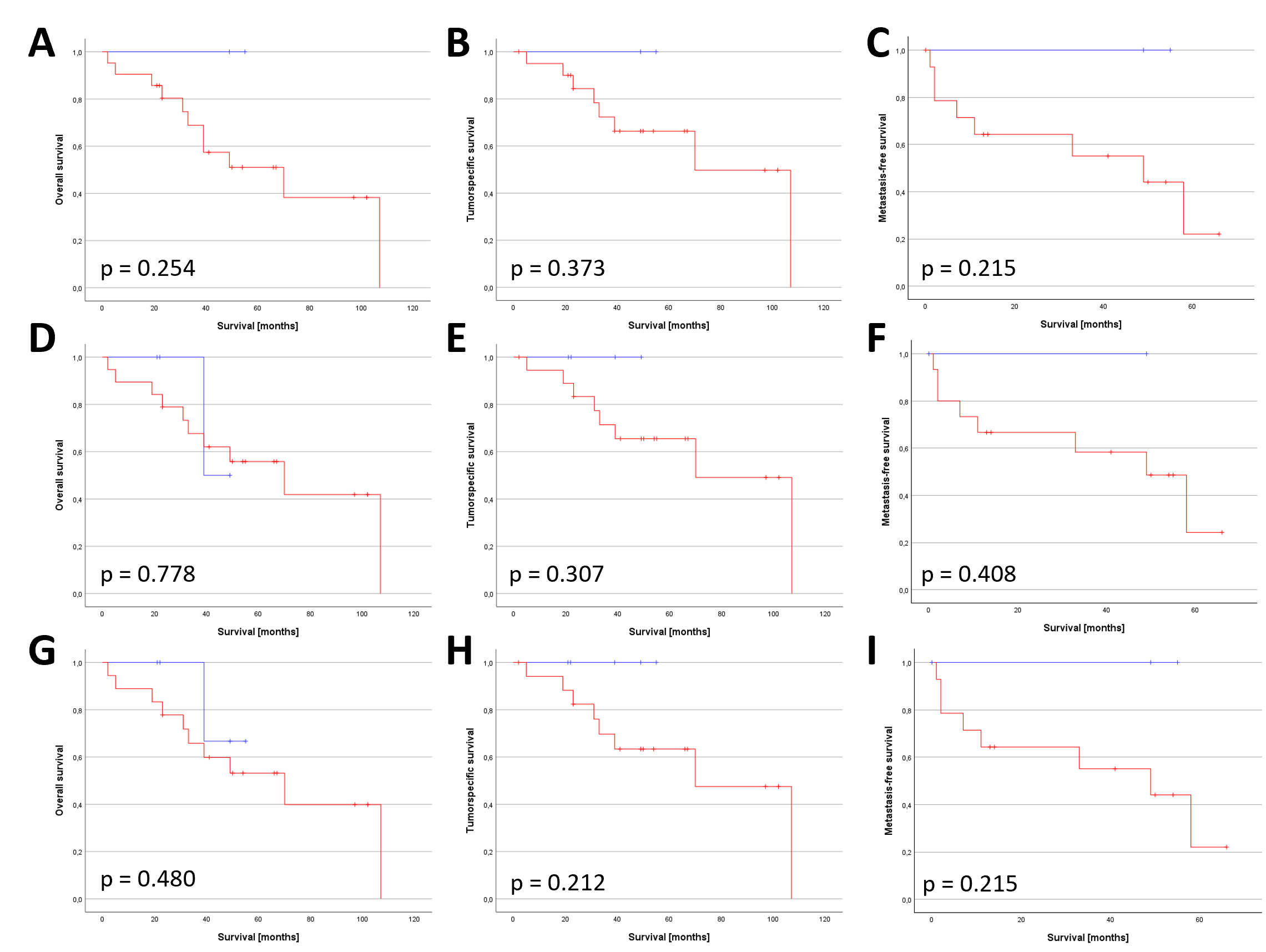


**Supplementary Figure 5: Kaplan-Meiers survival estimates of HPV- PeCa specimens for DKK1 alone and combined with p63 and CD15:** Kaplan-Meier survival estimates of DKK1+ (A-C), DKK1+CD15+ (D-F), DKK1+p63+ (G-I) and DKK1+p63+CD15+ (J-L) HPV- PeCa specimens for OS (A, D, G, J), TSS (B, E, H, K) and MFS (C, F, I, L). Estimates were calculated using Log-rank test. Red lines = positive, blue lines = negative.


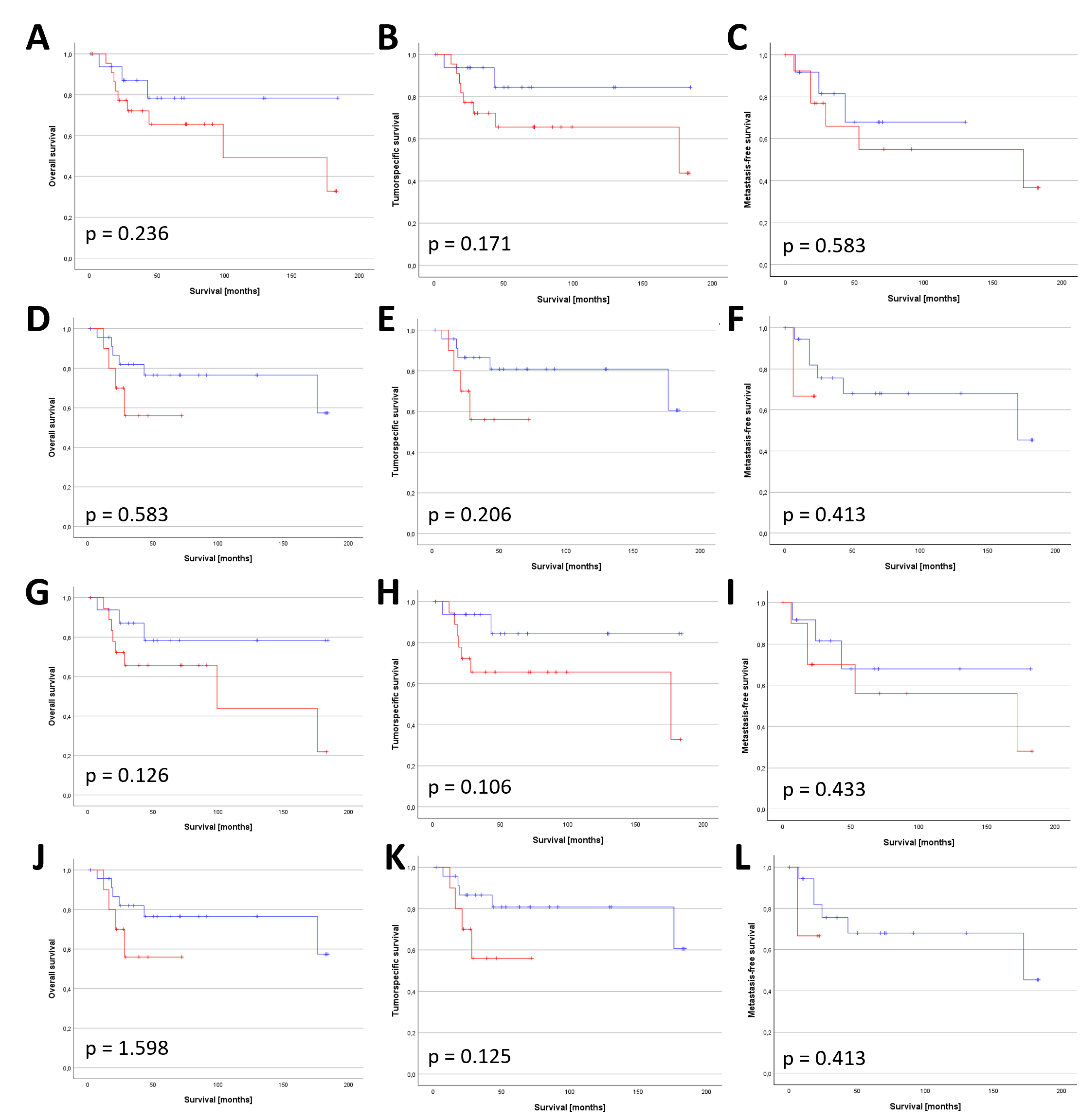


**Supplementary Figure 6: Kaplan-Meiers survival estimates of HPV+ PeCa specimens for DKK1 alone and combined with p63 and CD15:** Kaplan-Meier survival estimates of DKK1+ (A-C), DKK1+CD15+ (D-F), DKK1+p63+ (G-I) and DKK1+p63+CD15+ (J-L) HPV+ PeCa specimens for OS (A, D, G, J), TSS (B, E, H, K) and MFS (C, F, I, L). Estimates were calculated using Log-rank test. Red lines = positive, blue lines = negative.


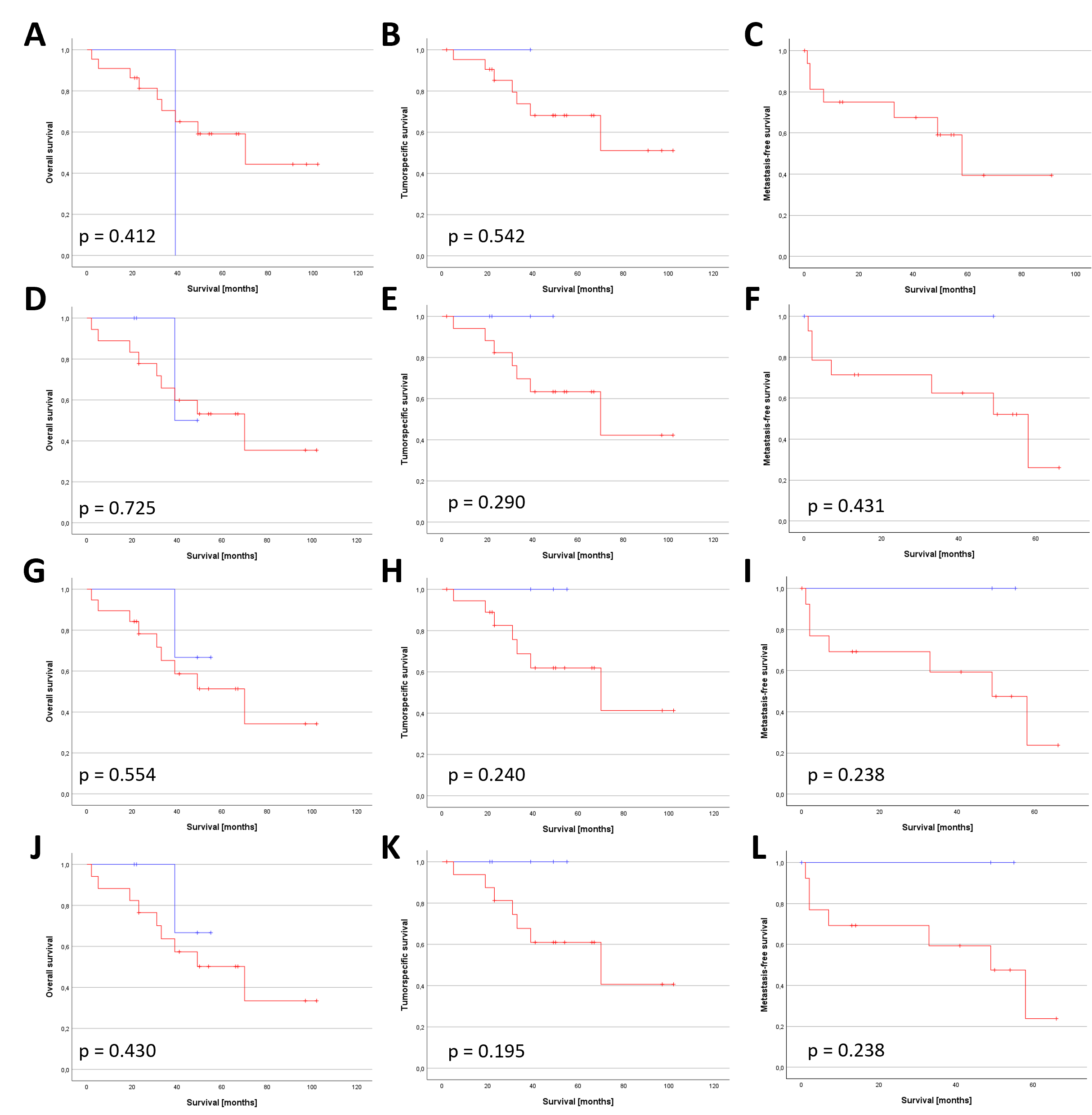


**Supplementary Figure 7: Kaplan-Meiers survival estimates of HPV- PeCa specimens for CD147 and CD15:** Kaplan-Meier survival estimates of CD147+ (A-C) and CD147+CD15+ (D-F) HPV- PeCa specimens for OS (A, D), TSS (B, E) and MFS (C, F). Estimates were calculated using Log-rank test. Red lines = positive, blue lines = negative.


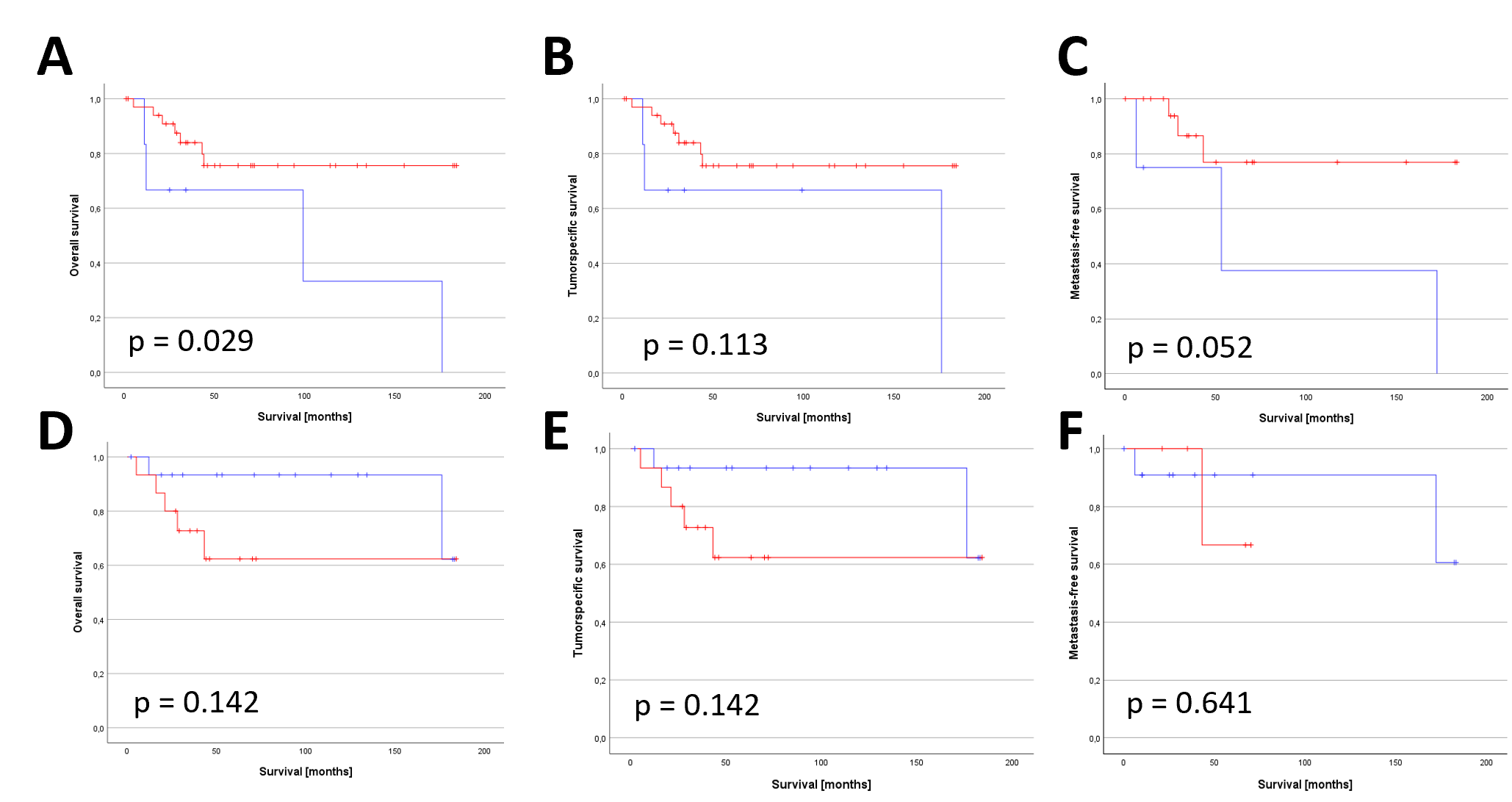


**Supplementary Figure 8: Kaplan-Meiers survival estimates of HPV+ PeCa specimens for CD147 and CD15:** Kaplan-Meier survival estimates of CD147+ (A-C) and CD147+CD15+ (D-F) HPV+ PeCa specimens for OS (A, D), TSS (B, E) and MFS (C, F). Estimates were calculated using Log-rank test. Red lines = positive, blue lines = negative.


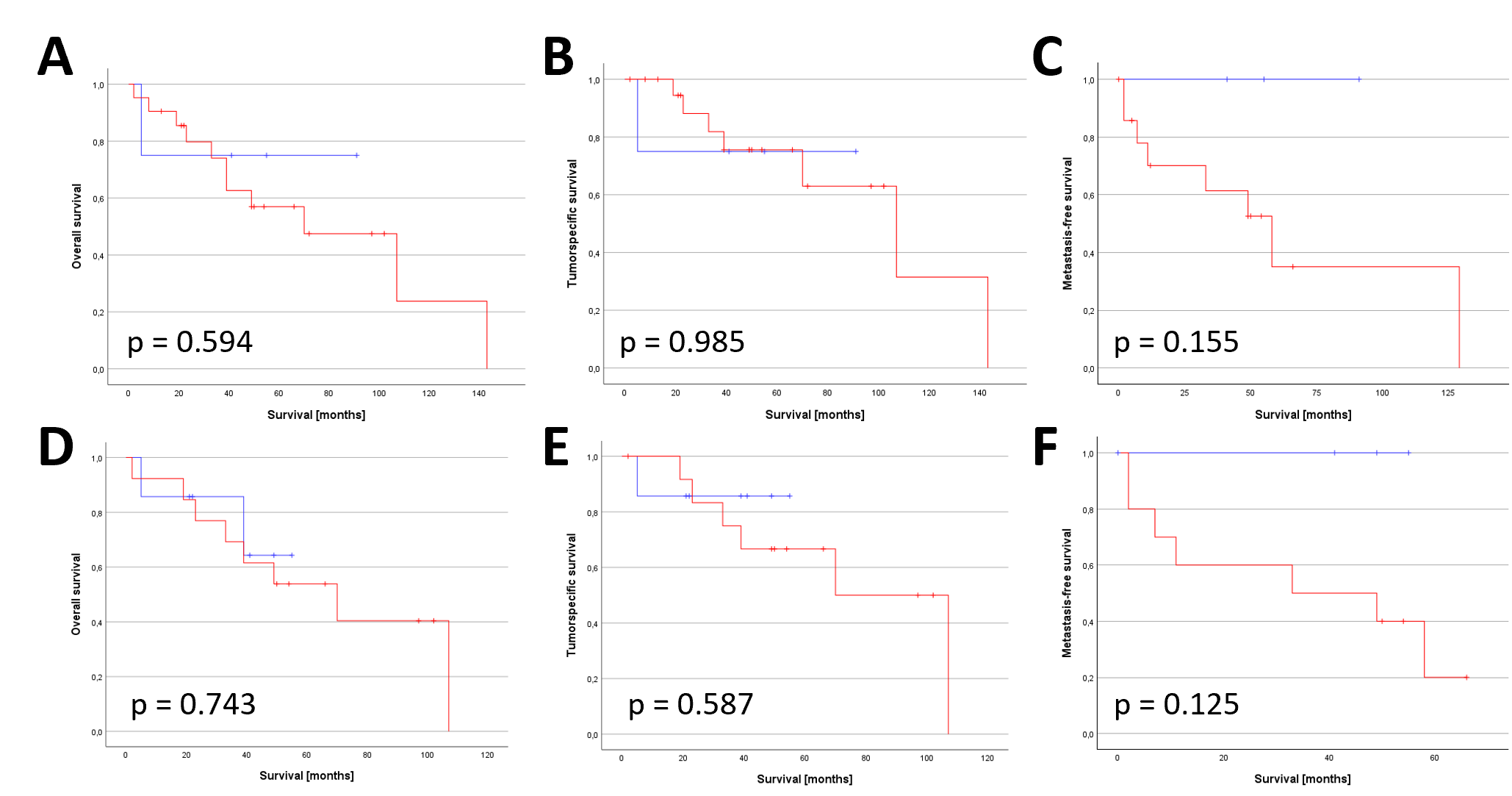


**Supplementary Table 3: Numbers-at-risk of HPV+ and HPV- PeCa cases**

| **Number-at-risk (HPV+ PeCa)** | |  | **Intervalls = 20 months** | | | | | | | | | |
| --- | --- | --- | --- | --- | --- | --- | --- | --- | --- | --- | --- | --- |
| Parameter |  | Survival | 0 | 20 | 40 | 60 | 80 | 100 | 120 | 140 | 160 | 180 |
| p63+ | negative | OS | 2 | 2 | 1 |  |  |  |  |  |  |  |
|  | positive |  | 21 | 17 | 9 | 5 | 3 | 2 |  |  |  |  |
|  | ∑ = sum |  | 23 | 19 | 10 | 5 | 3 | 2 | 0 | 0 | 0 | 0 |
|  | negative | TSS | 2 | 2 | 1 |  |  |  |  |  |  |  |
|  | positive |  | 21 | 16 | 8 | 5 | 3 | 2 |  |  |  |  |
|  | ∑ |  | 23 | 18 | 9 | 5 | 3 | 2 | 0 | 0 | 0 | 0 |
|  | negative | MFS | 2 | 2 | 1 |  |  |  |  |  |  |  |
|  | positive |  | 14 | 7 | 5 | 1 |  |  |  |  |  |  |
|  | ∑ |  | 16 | 9 | 6 | 1 | 0 | 0 | 0 | 0 | 0 | 0 |
| CD15+ | negative | OS | 4 | 3 | 1 |  |  |  |  |  |  |  |
|  | positive |  | 19 | 16 | 9 | 5 | 3 | 2 |  |  |  |  |
|  | ∑ |  | 23 | 19 | 10 | 5 | 3 | 2 | 0 | 0 | 0 | 0 |
|  | negative | TSS | 4 | 3 | 1 |  |  |  |  |  |  |  |
|  | positive |  | 19 | 16 | 9 | 5 | 3 | 2 |  |  |  |  |
|  | ∑ |  | 23 | 19 | 10 | 5 | 3 | 2 | 0 | 0 | 0 | 0 |
|  | negative | MFS | 2 | 1 | 1 |  |  |  |  |  |  |  |
|  | positive |  | 14 | 8 | 5 | 1 |  |  |  |  |  |  |
|  | ∑ |  | 16 | 9 | 6 | 1 | 0 | 0 | 0 | 0 | 0 | 0 |
| p63+CD15+ | negative | OS | 5 | 4 | 1 |  |  |  |  |  |  |  |
|  | positive |  | 18 | 15 | 9 | 5 | 3 | 2 |  |  |  |  |
|  | ∑ |  | 23 | 19 | 10 | 5 | 3 | 2 | 0 | 0 | 0 | 0 |
|  | negative | TSS | 5 | 4 | 1 |  |  |  |  |  |  |  |
|  | positive |  | 18 | 15 | 8 | 5 | 3 | 2 |  |  |  |  |
|  | ∑ |  | 23 | 19 | 9 | 5 | 3 | 2 | 0 | 0 | 0 | 0 |
|  | negative | MFS | 3 | 2 | 1 |  |  |  |  |  |  |  |
|  | positive |  | 13 | 7 | 5 | 1 |  |  |  |  |  |  |
|  | ∑ |  | 16 | 9 | 6 | 1 | 0 | 0 | 0 | 0 | 0 | 0 |
| DKK1+ | negative | OS | 1 | 1 |  |  |  |  |  |  |  |  |
|  | positive |  | 22 | 18 | 10 | 5 | 2 | 1 |  |  |  |  |
|  | ∑ = sum |  | 23 | 19 | 10 | 5 | 2 | 1 | 0 | 0 | 0 | 0 |
|  | negative | TSS | 1 | 1 |  |  |  |  |  |  |  |  |
|  | positive |  | 22 | 18 | 9 | 5 | 2 | 1 |  |  |  |  |
|  | ∑ |  | 23 | 19 | 9 | 5 | 2 | 1 | 0 | 0 | 0 | 0 |
|  | negative | MFS |  |  |  |  |  |  |  |  |  |  |
|  | positive |  | 16 | 10 | 7 | 2 | 1 |  |  |  |  |  |
|  | ∑ |  | 16 | 10 | 7 | 2 | 1 | 0 | 0 | 0 | 0 | 0 |
| DKK1+CD15+ | negative | OS | 4 | 3 | 1 |  |  |  |  |  |  |  |
|  | positive |  | 18 | 15 | 8 | 4 | 2 | 1 |  |  |  |  |
|  | ∑ |  | 22 | 18 | 9 | 4 | 2 | 1 | 0 | 0 | 0 | 0 |
|  | negative | TSS | 4 | 3 | 1 |  |  |  |  |  |  |  |
|  | positive |  | 18 | 15 | 8 | 4 | 2 | 1 |  |  |  |  |
|  | ∑ |  | 22 | 18 | 9 | 4 | 2 | 1 | 0 | 0 | 0 | 0 |
|  | negative | MFS | 1 | 0 | 1 |  |  |  |  |  |  |  |
|  | positive |  | 2 | 0 | 4 | 1 |  |  |  |  |  |  |
|  | ∑ |  | 3 | 0 | 5 | 1 | 0 | 0 | 0 | 0 | 0 | 0 |
| DKK1+p63+ CD15+ | negative | OS | 5 | 4 | 1 |  |  |  |  |  |  |  |
|  | positive |  | 17 | 14 | 8 | 4 | 2 | 1 |  |  |  |  |
|  | ∑ |  | 22 | 18 | 9 | 4 | 2 | 1 | 0 | 0 | 0 | 0 |
|  | negative | TSS | 5 | 4 | 1 |  |  |  |  |  |  |  |
|  | positive |  | 17 | 14 | 7 | 4 | 2 | 1 |  |  |  |  |
|  | ∑ |  | 22 | 18 | 8 | 4 | 2 | 1 | 0 | 0 | 0 | 0 |
|  | negative | MFS | 3 | 2 | 1 |  |  |  |  |  |  |  |
|  | positive |  | 12 | 7 | 5 | 1 |  |  |  |  |  |  |
|  | ∑ |  | 15 | 9 | 6 | 1 | 0 | 0 | 0 | 0 | 0 | 0 |
| DKK1+p63+ | negative | OS | 3 | 3 | 1 |  |  |  |  |  |  |  |
|  | positive |  | 19 | 15 | 8 | 4 | 2 | 1 |  |  |  |  |
|  | ∑ |  | 22 | 18 | 9 | 4 | 2 | 1 | 0 | 0 | 0 | 0 |
|  | negative | TSS | 3 | 3 | 1 |  |  |  |  |  |  |  |
|  | positive |  | 19 | 15 | 7 | 4 | 2 | 1 |  |  |  |  |
|  | ∑ |  | 22 | 18 | 8 | 4 | 2 | 1 | 0 | 0 | 0 | 0 |
|  | negative | MFS | 0 | 0 | 2 |  |  |  |  |  |  |  |
|  | positive |  | 3 | 0 | 3 | 1 |  |  |  |  |  |  |
|  | ∑ |  | 3 | 0 | 5 | 1 | 0 | 0 | 0 | 0 | 0 | 0 |
| CD147+ | negative | OS | 4 | 3 | 2 | 1 | 1 |  |  |  |  |  |
|  | positive |  | 21 | 16 | 10 | 6 | 4 | 3 | 1 | 1 |  |  |
|  | ∑ |  | 25 | 19 | 12 | 7 | 4 | 3 | 1 | 1 | 0 | 0 |
|  | negative | TSS | 4 | 3 | 2 | 1 | 1 |  |  |  |  |  |
|  | positive |  | 20 | 16 | 9 | 6 | 4 | 3 | 1 | 1 |  |  |
|  | ∑ |  | 24 | 19 | 11 | 7 | 4 | 3 | 1 | 1 | 0 | 0 |
|  | negative | MFS | 3 | 3 | 2 | 1 | 1 |  |  |  |  |  |
|  | positive |  | 14 | 8 | 6 | 2 | 1 | 1 | 1 |  |  |  |
|  | ∑ |  | 17 | 11 | 8 | 3 | 2 | 1 | 1 | 0 | 0 | 0 |
| CD147+CD15+ | negative | OS | 7 | 5 | 2 |  |  |  |  |  |  |  |
|  | positive |  | 13 | 11 | 7 | 5 | 3 | 2 |  |  |  |  |
|  | ∑ |  | 20 | 16 | 9 | 5 | 3 | 2 | 0 | 0 | 0 | 0 |
|  | negative | TSS | 7 | 5 | 2 |  |  |  |  |  |  |  |
|  | positive |  | 13 | 11 | 7 | 5 | 3 | 2 |  |  |  |  |
|  | ∑ |  | 20 | 16 | 9 | 5 | 3 | 2 | 0 | 0 | 0 | 0 |
|  | negative | MFS | 4 | 3 | 2 |  |  |  |  |  |  |  |
|  | positive |  | 10 | 6 | 4 | 1 |  |  |  |  |  |  |
|  | ∑ |  | 14 | 9 | 6 | 1 | 0 | 0 | 0 | 0 | 0 | 0 |
|  | |  |  | | | | | | | | | |
| **Number-at-risk (HPV- PeCa)** | |  | **Intervalls = 20 months** | | | | | | | | | |
| **Parameter** |  | **Survival** | **0** | **20** | **40** | **60** | **80** | **100** | **120** | **140** | **160** | **180** |
| p63+ | negative | OS | 4 | 4 | 2 | 1 | 1 | 1 | 1 | 1 | 1 | 1 |
|  | positive |  | 36 | 25 | 17 | 13 | 10 | 7 | 5 | 3 | 3 | 1 |
|  | ∑ = sum |  | 40 | 29 | 19 | 14 | 11 | 8 | 6 | 4 | 4 | 2 |
|  | negative | TSS | 4 | 4 | 2 | 1 | 1 | 1 | 1 | 1 | 1 | 1 |
|  | positive |  | 36 | 24 | 17 | 13 | 9 | 7 | 5 | 3 | 3 | 1 |
|  | ∑ |  | 40 | 28 | 19 | 14 | 10 | 8 | 6 | 4 | 4 | 2 |
|  | negative | MFS | 3 | 3 | 2 | 1 | 1 | 1 | 1 | 1 | 1 | 1 |
|  | positive |  | 21 | 13 | 9 | 6 | 4 | 3 | 3 | 2 | 2 | 1 |
|  | ∑ |  | 24 | 16 | 11 | 7 | 5 | 4 | 4 | 3 | 3 | 1 |
| CD15 | negative | OS | 19 | 14 | 11 | 10 | 8 | 6 | 4 | 3 | 3 | 1 |
|  | positive |  | 20 | 14 | 7 | 4 | 2 | 2 | 2 | 1 | 1 | 1 |
|  | ∑ |  | 39 | 28 | 18 | 14 | 10 | 8 | 6 | 4 | 4 | 2 |
|  | negative | TSS | 19 | 13 | 11 | 10 | 8 | 6 | 4 | 3 | 3 | 1 |
|  | positive |  | 20 | 14 | 7 | 4 | 2 | 2 | 2 | 1 | 1 | 1 |
|  | ∑ |  | 39 | 27 | 18 | 14 | 10 | 8 | 6 | 4 | 4 | 2 |
|  | negative | MFS | 14 | 9 | 6 | 5 | 4 | 3 | 3 | 3 | 3 | 1 |
|  | positive |  | 9 | 6 | 4 | 2 | 1 | 1 | 1 |  |  |  |
|  | ∑ |  | 23 | 15 | 10 | 7 | 5 | 4 | 4 | 3 | 3 | 1 |
| p63+CD15+ | negative | OS | 19 | 14 | 11 | 10 | 8 | 6 | 4 | 3 | 3 | 1 |
|  | positive |  | 20 | 14 | 7 | 4 | 2 | 2 | 2 | 1 | 1 | 1 |
|  | ∑ |  | 39 | 28 | 18 | 14 | 10 | 8 | 6 | 4 | 4 | 2 |
|  | negative | TSS | 19 | 13 | 11 | 10 | 8 | 6 | 4 | 3 | 3 | 1 |
|  | positive |  | 20 | 14 | 7 | 4 | 2 | 2 | 2 | 1 | 1 | 1 |
|  | ∑ |  | 39 | 27 | 18 | 14 | 10 | 8 | 6 | 4 | 4 | 2 |
|  | negative | MFS | 14 | 9 | 6 | 5 | 4 | 3 | 3 | 3 | 3 | 1 |
|  | positive |  | 9 | 6 | 4 | 2 | 1 | 1 | 1 |  |  |  |
|  | ∑ |  | 23 | 15 | 10 | 7 | 5 | 4 | 4 | 3 | 3 | 1 |
| DKK1+ | negative | OS | 16 | 13 | 9 | 5 | 3 | 3 | 2 | 1 | 1 | 1 |
|  | positive |  | 23 | 16 | 11 | 8 | 5 | 3 | 3 | 3 | 3 | 1 |
|  | ∑ = sum |  | 39 | 29 | 20 | 13 | 8 | 6 | 5 | 4 | 4 | 2 |
|  | negative | TSS | 16 | 12 | 9 | 5 | 3 | 3 | 2 | 1 | 1 | 1 |
|  | positive |  | 23 | 16 | 11 | 8 | 5 | 3 | 3 | 3 | 3 | 1 |
|  | ∑ |  | 39 | 28 | 20 | 13 | 8 | 6 | 5 | 4 | 4 | 2 |
|  | negative | MFS | 11 | 8 | 6 | 3 | 1 | 1 | 1 |  |  |  |
|  | positive |  | 14 | 9 | 6 | 5 | 4 | 3 | 3 | 3 | 3 | 1 |
|  | ∑ |  | 25 | 17 | 12 | 7 | 5 | 4 | 4 | 3 | 3 | 1 |
| DKK1+CD15+ | negative | OS | 23 | 18 | 14 | 10 | 7 | 6 | 5 | 4 | 4 | 2 |
|  | positive |  | 10 | 6 | 2 | 1 |  |  |  |  |  |  |
|  | ∑ |  | 33 | 24 | 16 | 11 | 7 | 6 | 5 | 4 | 4 | 2 |
|  | negative | TSS | 23 | 17 | 14 | 10 | 7 | 6 | 5 | 4 | 4 | 2 |
|  | positive |  | 10 | 6 | 2 | 1 |  |  |  |  |  |  |
|  | ∑ |  | 33 | 23 | 16 | 11 | 7 | 6 | 5 | 4 | 4 | 2 |
|  | negative | MFS | 18 | 12 | 10 | 7 | 5 | 4 | 4 | 3 | 3 | 1 |
|  | positive |  | 3 | 1 |  |  |  |  |  |  |  |  |
|  | ∑ |  | 21 | 13 | 10 | 7 | 5 | 4 | 4 | 3 | 3 | 1 |
| DKK1+p63+ CD15+ | negative | OS | 23 | 18 | 14 | 10 | 7 | 6 | 5 | 4 | 4 | 2 |
|  | positive |  | 10 | 6 | 2 | 1 |  |  |  |  |  |  |
|  | ∑ |  | 33 | 24 | 16 | 11 | 7 | 6 | 5 | 4 | 4 | 2 |
|  | negative | TSS | 23 | 17 | 14 | 10 | 7 | 6 | 5 | 4 | 4 | 2 |
|  | positive |  | 10 | 6 | 2 | 1 |  |  |  |  |  |  |
|  | ∑ |  | 33 | 23 | 16 | 11 | 7 | 6 | 5 | 4 | 4 | 2 |
|  | negative | MFS | 18 | 12 | 10 | 7 | 5 | 4 | 4 | 3 | 3 | 1 |
|  | positive |  | 3 | 1 |  |  |  |  |  |  |  |  |
|  | ∑ |  | 21 | 13 | 10 | 7 | 5 | 4 | 4 | 3 | 3 | 1 |
| DKK1+p63+ | negative | OS | 16 | 13 | 9 | 5 | 4 | 4 | 3 | 2 | 2 | 1 |
|  | positive |  | 19 | 12 | 8 | 6 | 4 | 2 | 2 | 2 | 2 | 1 |
|  | ∑ |  | 35 | 25 | 17 | 11 | 8 | 6 | 5 | 4 | 4 | 2 |
|  | negative | TSS | 16 | 12 | 9 | 5 | 4 | 4 | 3 | 2 | 2 | 1 |
|  | positive |  | 19 | 12 | 8 | 6 | 4 | 2 | 2 | 2 | 2 | 1 |
|  | ∑ |  | 35 | 24 | 17 | 11 | 8 | 6 | 5 | 4 | 4 | 2 |
|  | negative | MFS | 11 | 8 | 6 | 3 | 2 | 2 | 2 | 1 | 1 | 1 |
|  | positive |  | 11 | 6 | 5 | 4 | 3 | 2 | 2 | 2 | 2 | 1 |
|  | ∑ |  | 22 | 14 | 11 | 7 | 5 | 4 | 4 | 3 | 3 | 1 |
| CD147+ | negative | OS | 6 | 3 | 2 | 2 | 2 | 1 | 1 | 1 | 1 |  |
|  | positive |  | 34 | 27 | 18 | 12 | 9 | 7 | 5 | 4 | 3 | 2 |
|  | ∑ |  | 40 | 30 | 20 | 14 | 11 | 8 | 6 | 5 | 4 | 2 |
|  | negative | TSS | 6 | 3 | 2 | 2 | 2 | 1 | 1 | 1 | 1 |  |
|  | positive |  | 34 | 27 | 18 | 12 | 9 | 7 | 5 | 4 | 3 | 2 |
|  | ∑ |  | 40 | 30 | 20 | 14 | 11 | 8 | 6 | 5 | 4 | 2 |
|  | negative | MFS | 4 | 2 | 2 | 1 | 1 | 1 | 1 | 1 | 1 |  |
|  | positive |  | 19 | 14 | 9 | 6 | 4 | 4 | 3 | 3 | 2 | 1 |
|  | ∑ |  | 23 | 16 | 11 | 7 | 5 | 5 | 4 | 4 | 3 | 1 |
| CD147+CD15+ | negative | OS | 15 | 12 | 10 | 9 | 7 | 6 | 4 | 3 | 3 | 1 |
|  | positive |  | 15 | 11 | 6 | 3 | 1 | 1 | 1 | 1 | 1 | 1 |
|  | ∑ |  | 30 | 23 | 16 | 12 | 8 | 7 | 5 | 4 | 4 | 2 |
|  | negative | TSS | 15 | 12 | 10 | 9 | 7 | 6 | 4 | 3 | 3 | 1 |
|  | positive |  | 15 | 11 | 6 | 3 | 1 | 1 | 1 | 1 | 1 | 1 |
|  | ∑ |  | 30 | 23 | 16 | 12 | 8 | 7 | 5 | 4 | 4 | 2 |
|  | negative | MFS | 11 | 7 | 5 | 4 | 3 | 3 | 3 | 3 | 3 | 1 |
|  | positive |  | 5 | 4 | 3 | 1 |  |  |  |  |  |  |
|  | ∑ |  | 16 | 11 | 8 | 5 | 3 | 3 | 3 | 3 | 3 | 1 |

**Supplementary Table 4: Sample sizes required for robust statistics:** The sample sizes were calculated using the powerSurvEpi R package and the ssizeCT function based on the survival data and the HR of Table 3 using an α adjusted for multiple comparisons (0.0019) and a power of 0.8. The final calculated total sample sizes reflect the prevalence of each parameter within the overall cohort and estimate the number of tumor samples required to calculate a significant result regarding the impact of each parameter on survival.

**Supplementary Table 5: Patient characteristics:**

|  | Total number (n) | Percentage (%) |
| --- | --- | --- |
| Patients | 94 | |
| Age | | |
| Minimum | 24 |  |
| Maximum | 88 |  |
| <50 | 22 | 23.4 |
| 51-70 | 48 | 51.1 |
| >70 | 19 | 20.2 |
| Median Age (95% CI) | 60 +/- 14.6 (56.9-63.1) | |
| Tumor Expansion | | |
| Non-invasive (pTis, pT1a) | 30 | 31.9 |
| Invasive (pT1b-4) | 55 | 58.5 |
| Involved Lymph nodes | | |
| Non-metastasized (cN0, pN0) | 58 | 61.7 |
| Metastasized (pN1-3) | 27 | 28.7 |
| Distant Metastasis | | |
| M | 4 | 4.3 |
| Grading | | |
| well differentiated (1) | 16 | 17.0 |
| moderately differentiated (2) | 53 | 56.8 |
| poorly differentiated (3) | 25 | 26.6 |
| HPV Status | | |
| HPV+ | 33 | 35.1 |
| HPV- | 61 | 64.9 |
| Histological subtypes | | |
| 1 usual type SCC | 36 | 38.3 |
| 2 Pseudohyperplastic carcinoma | 7 | 7.45 |
| 3 Pseudoglandular carcinoma | 1 | 1.06 |
| 4 Pure verrucous carcinoma | 6 | 6.38 |
| 5 Carcinoma cunicolatum | 1 | 1.06 |
| 6 Papillary carcinoma (NOS) | 2 | 2.13 |
| 10 Papillary-basaloid carcinoma | 1 | 1.06 |
| 11 Warty-basaloid carcinoma | 14 | 14.89 |
| 12 Warty carcinoma | 10 | 10.68 |
| 13 Basaloid carcinoma | 15 | 15.96 |
| 14 Clear cell carcinoma | 1 | 1.06 |
| Therapy | | |
| Chemotherapy | 19 | 20.21 |
| Radiotherapy | 5 | 5.32 |
| Surgical procedure | 62 | 65.96 |

**Supplementary Table 6: Numbers-at-risk for all evaluated parameters**

| Number-at-risk |  |  | Intervalls = 20 months | | | | | | | | | |
| --- | --- | --- | --- | --- | --- | --- | --- | --- | --- | --- | --- | --- |
| Parameter |  | **survival** | **0** | **20** | **40** | **60** | **80** | **100** | **120** | **140** | **160** | **180** |
| p63 | negative | OS | 6 | 6 | 3 | 1 | 1 | 1 | 1 | 1 | 1 | 1 |
|  | positive |  | 56 | 42 | 26 | 18 | 12 | 8 | 5 | 3 | 3 | 1 |
|  | ∑ = sum |  | 62 | 48 | 29 | 19 | 13 | 9 | 6 | 4 | 4 | 2 |
|  | negative | TSS | 6 | 6 | 3 | 1 | 1 | 1 | 1 | 1 | 1 | 1 |
|  | positive |  | 56 | 40 | 25 | 18 | 12 | 8 | 5 | 3 | 3 | 1 |
|  | ∑ |  | 62 | 46 | 28 | 19 | 13 | 9 | 6 | 4 | 4 | 2 |
|  | negative | MFS | 5 | 5 | 3 | 1 | 1 | 1 | 1 | 1 | 1 | 1 |
|  | positive |  | 34 | 20 | 14 | 6 | 4 | 3 | 3 | 2 | 2 | 1 |
|  | ∑ |  | 39 | 25 | 17 | 7 | 5 | 4 | 4 | 3 | 3 | 1 |
| p63+CD15+ | negative | OS | 24 | 18 | 12 | 10 | 8 | 6 | 4 | 3 | 3 | 1 |
|  | positive |  | 38 | 29 | 16 | 9 | 5 | 4 | 2 | 1 | 1 | 1 |
|  | ∑ |  | 62 | 47 | 28 | 19 | 13 | 10 | 6 | 4 | 4 | 2 |
|  | negative | TSS | 24 | 17 | 12 | 10 | 8 | 6 | 4 | 3 | 3 | 1 |
|  | positive |  | 38 | 28 | 15 | 9 | 5 | 4 | 2 | 1 | 1 | 1 |
|  | ∑ |  | 62 | 45 | 27 | 19 | 13 | 10 | 6 | 4 | 4 | 2 |
|  | negative | MFS | 17 | 11 | 7 | 5 | 4 | 3 | 3 | 3 | 3 | 1 |
|  | positive |  | 22 | 13 | 9 | 3 | 1 | 1 | 1 |  |  |  |
|  | ∑ |  | 39 | 24 | 16 | 8 | 5 | 4 | 4 | 3 | 3 | 1 |
| CD15 | negative | OS | 23 | 17 | 12 | 10 | 8 | 6 | 4 | 3 | 3 | 1 |
|  | positive |  | 39 | 30 | 16 | 9 | 5 | 4 | 2 | 1 | 1 | 1 |
|  | ∑ |  | 62 | 47 | 28 | 19 | 13 | 10 | 6 | 4 | 4 | 2 |
|  | negative | TSS | 23 | 16 | 12 | 10 | 8 | 6 | 4 | 3 | 3 | 1 |
|  | positive |  | 39 | 29 | 16 | 9 | 5 | 4 | 2 | 1 | 1 | 1 |
|  | ∑ |  | 62 | 45 | 28 | 19 | 13 | 10 | 6 | 4 | 4 | 2 |
|  | negative | MFS | 16 | 10 | 6 | 5 | 4 | 3 | 3 | 3 | 3 | 1 |
|  | positive |  | 23 | 14 | 9 | 3 | 1 | 1 | 1 |  |  |  |
|  | ∑ |  | 39 | 24 | 15 | 8 | 5 | 4 | 4 | 3 | 3 | 1 |
| HPV | negative | OS | 53 | 39 | 26 | 18 | 14 | 10 | 8 | 5 | 4 | 2 |
|  | positive |  | 31 | 25 | 15 | 10 | 6 | 5 | 3 | 3 | 1 | 1 |
|  | ∑ |  | 84 | 64 | 41 | 28 | 20 | 15 | 11 | 8 | 5 | 2 |
|  | negative | TSS | 53 | 38 | 26 | 18 | 13 | 10 | 8 | 5 | 4 | 2 |
|  | positive |  | 30 | 24 | 15 | 10 | 6 | 5 | 3 | 3 | 1 | 1 |
|  | ∑ |  | 83 | 62 | 41 | 28 | 19 | 15 | 11 | 8 | 5 | 2 |
|  | negative | MFS | 33 | 22 | 15 | 11 | 8 | 7 | 6 | 4 | 3 | 1 |
|  | positive |  | 20 | 13 | 9 | 4 | 3 | 2 | 2 | 1 |  |  |
|  | ∑ |  | 53 | 35 | 24 | 15 | 11 | 9 | 8 | 5 | 3 | 1 |
| HPV+p63+ | negative | OS | 42 | 31 | 20 | 14 | 11 | 8 | 6 | 4 | 4 | 2 |
|  | positive |  | 21 | 17 | 9 | 5 | 3 | 2 |  |  |  |  |
|  | ∑ |  | 63 | 48 | 29 | 19 | 14 | 10 | 6 | 4 | 4 | 2 |
|  | negative | TSS | 42 | 30 | 20 | 14 | 10 | 8 | 6 | 4 | 4 | 2 |
|  | positive |  | 21 | 16 | 8 | 5 | 3 | 2 |  |  |  |  |
|  | ∑ |  | 63 | 46 | 28 | 19 | 13 | 10 | 6 | 4 | 4 | 2 |
|  | negative | MFS | 26 | 17 | 12 | 7 | 5 | 4 | 4 | 3 | 3 | 1 |
|  | positive |  | 14 | 7 | 5 | 1 |  |  |  |  |  |  |
|  | ∑ |  | 40 | 24 | 17 | 8 | 5 | 4 | 4 | 3 | 3 | 1 |
| HPV+CD15+ | negative | OS | 43 | 31 | 19 | 13 | 10 | 8 | 6 | 4 | 4 | 2 |
|  | positive |  | 19 | 16 | 9 | 5 | 3 | 2 |  |  |  |  |
|  | ∑ |  | 62 | 47 | 28 | 18 | 13 | 10 | 6 | 4 | 4 | 2 |
|  | negative | TSS | 43 | 29 | 19 | 13 | 10 | 8 | 6 | 4 | 4 | 2 |
|  | positive |  | 19 | 16 | 9 | 5 | 3 | 2 |  |  |  |  |
|  | ∑ |  | 62 | 45 | 28 | 18 | 13 | 10 | 6 | 4 | 4 | 2 |
|  | negative | MFS | 24 | 15 | 10 | 7 | 5 | 4 | 4 | 3 | 3 | 1 |
|  | positive |  | 14 | 8 | 5 | 1 |  |  |  |  |  |  |
|  | ∑ |  | 38 | 23 | 15 | 8 | 5 | 4 | 4 | 3 | 3 | 1 |
| HPV+p63+CD15+ | negative | OS | 44 | 32 | 19 | 13 | 10 | 8 | 6 | 4 | 4 | 2 |
|  | positive |  | 18 | 15 | 9 | 5 | 3 | 2 |  |  |  |  |
|  | ∑ |  | 62 | 47 | 28 | 18 | 13 | 10 | 6 | 4 | 4 | 2 |
|  | negative | TSS | 44 | 30 | 19 | 13 | 10 | 8 | 6 | 4 | 4 | 2 |
|  | positive |  | 18 | 15 | 8 | 5 | 3 | 2 |  |  |  |  |
|  | ∑ |  | 62 | 45 | 28 | 18 | 13 | 10 | 6 | 4 | 4 | 2 |
|  | negative | MFS | 25 | 16 | 11 | 7 | 5 | 4 | 4 | 3 | 3 | 1 |
|  | positive |  | 13 | 7 | 5 | 1 |  |  |  |  |  |  |
|  | ∑ |  | 38 | 23 | 16 | 8 | 5 | 4 | 4 | 3 | 3 | 1 |
| DKK1+ | negative | OS | 17 | 14 | 9 | 5 | 3 | 3 | 2 | 1 | 1 | 1 |
|  | positive |  | 45 | 33 | 20 | 13 | 7 | 4 | 3 | 3 | 3 | 1 |
|  | ∑ |  | 62 | 47 | 29 | 18 | 17 | 7 | 5 | 4 | 4 | 2 |
|  | negative | TSS | 17 | 13 | 9 | 5 | 3 | 3 | 2 | 1 | 1 | 1 |
|  | positive |  | 44 | 33 | 20 | 13 | 7 | 4 | 3 | 3 | 3 | 1 |
|  | ∑ |  | 61 | 46 | 29 | 18 | 10 | 7 | 5 | 4 | 4 | 2 |
|  | negative | MFS | 11 | 8 | 6 | 3 | 1 | 1 | 1 |  |  |  |
|  | positive |  | 29 | 19 | 13 | 6 | 4 | 3 | 3 | 3 | 3 | 1 |
|  | ∑ |  | 40 | 27 | 19 | 9 | 5 | 4 | 4 | 3 | 3 | 1 |
| HPV+DKK1+ | negative | OS | 40 | 29 | 19 | 12 | 8 | 6 | 5 | 4 | 4 | 2 |
|  | positive |  | 22 | 18 | 10 | 5 | 2 | 1 |  |  |  |  |
|  | ∑ |  | 62 | 47 | 29 | 17 | 10 | 7 | 5 | 4 | 4 | 2 |
|  | negative | TSS | 40 | 28 | 19 | 12 | 8 | 6 | 5 | 4 | 4 | 2 |
|  | positive |  | 22 | 18 | 9 | 5 | 2 | 1 |  |  |  |  |
|  | ∑ |  | 62 | 46 | 28 | 17 | 10 | 7 | 5 | 4 | 4 | 2 |
|  | negative | MFS | 25 | 17 | 12 | 7 | 5 | 4 | 4 | 3 | 3 | 1 |
|  | positive |  | 16 | 10 | 7 | 2 | 1 |  |  |  |  |  |
|  | ∑ |  | 41 | 27 | 19 | 9 | 6 | 4 | 4 | 3 | 3 | 1 |
| DKK1+CD15+ | negative | OS | 27 | 21 | 14 | 10 | 7 | 6 | 5 | 4 | 4 | 2 |
|  | positive |  | 28 | 21 | 10 | 5 | 2 | 1 |  |  |  |  |
|  | ∑ |  | 55 | 42 | 24 | 15 | 9 | 7 | 5 | 4 | 4 | 2 |
|  | negative | TSS | 27 | 20 | 14 | 10 | 7 | 6 | 5 | 4 | 4 | 2 |
|  | positive |  | 28 | 21 | 9 | 5 | 2 | 1 |  |  |  |  |
|  | ∑ |  | 55 | 41 | 23 | 15 | 9 | 7 | 5 | 4 | 4 | 2 |
|  | negative | MFS | 19 | 13 | 10 | 7 | 5 | 4 | 4 | 3 | 3 | 1 |
|  | positive |  | 16 | 9 | 5 | 1 |  |  |  |  |  |  |
|  | ∑ |  | 35 | 22 | 15 | 8 | 5 | 4 | 4 | 3 | 3 | 1 |
| DKK1+p63+CD15+ | negative | OS | 28 | 22 | 15 | 10 | 7 | 6 | 5 | 4 | 4 | 2 |
|  | positive |  | 27 | 20 | 9 | 5 | 2 | 1 |  |  |  |  |
|  | ∑ |  | 55 | 42 | 24 | 15 | 9 | 7 | 5 | 4 | 4 | 2 |
|  | negative | TSS | 28 | 21 | 15 | 10 | 7 | 6 | 5 | 4 | 4 | 2 |
|  | positive |  | 27 | 20 | 9 | 5 | 2 | 1 |  |  |  |  |
|  | ∑ |  | 55 | 41 | 24 | 15 | 9 | 7 | 5 | 4 | 4 | 2 |
|  | negative | MFS | 20 | 14 | 11 | 7 | 5 | 4 | 4 | 3 | 3 | 1 |
|  | positive |  | 15 | 8 | 5 | 1 |  |  |  |  |  |  |
|  | ∑ |  | 35 | 22 | 16 | 8 | 5 | 4 | 4 | 3 | 3 | 1 |
| DKK1+p63+ | negative | OS | 19 | 16 | 10 | 5 | 4 | 4 | 3 | 2 | 2 | 1 |
|  | positive |  | 38 | 27 | 15 | 10 | 6 | 3 | 2 | 2 | 2 | 1 |
|  | ∑ |  | 57 | 43 | 25 | 15 | 10 | 7 | 5 | 4 | 4 | 2 |
|  | negative | TSS | 19 | 15 | 10 | 5 | 4 | 4 | 3 | 2 | 2 | 1 |
|  | positive |  | 37 | 27 | 15 | 10 | 5 | 3 | 2 | 2 | 2 | 1 |
|  | ∑ |  | 56 | 42 | 25 | 15 | 9 | 7 | 5 | 4 | 4 | 2 |
|  | negative | MFS | 13 | 10 | 7 | 3 | 2 | 2 | 2 | 1 | 1 | 1 |
|  | positive |  | 23 | 13 | 10 | 4 | 3 | 2 | 2 | 2 | 2 | 1 |
|  | ∑ |  | 36 | 23 | 17 | 7 | 5 | 4 | 4 | 3 | 3 | 1 |
| HPV+DKK1+ CD15+ | negative | OS | 37 | 27 | 16 | 10 | 7 | 6 | 5 | 4 | 4 | 2 |
|  | positive |  | 18 | 15 | 8 | 4 | 2 | 1 |  |  |  |  |
|  | ∑ |  | 55 | 42 | 24 | 14 | 9 | 7 | 5 | 4 | 4 | 2 |
|  | negative | TSS | 37 | 26 | 16 | 10 | 7 | 6 | 5 | 4 | 4 | 2 |
|  | positive |  | 18 | 15 | 8 | 4 | 2 | 1 |  |  |  |  |
|  | ∑ |  | 55 | 41 | 24 | 14 | 9 | 7 | 5 | 4 | 4 | 2 |
|  | negative | MFS | 22 | 14 | 10 | 7 | 5 | 4 | 4 | 3 | 3 | 1 |
|  | positive |  | 13 | 8 | 5 | 1 |  |  |  |  |  |  |
|  | ∑ |  | 35 | 22 | 15 | 7 | 5 | 4 | 4 | 3 | 3 | 1 |
| HPV+DKK1+ p63+CD15+ | negative | OS | 38 | 28 | 16 | 10 | 7 | 6 | 5 | 4 | 4 | 2 |
|  | positive |  | 17 | 14 | 8 | 4 | 2 | 1 |  |  |  |  |
|  | ∑ |  | 55 | 42 | 24 | 14 | 9 | 7 | 5 | 4 | 4 | 2 |
|  | negative | TSS | 38 | 27 | 16 | 10 | 7 | 6 | 5 | 4 | 4 | 2 |
|  | positive |  | 17 | 14 | 7 | 4 | 2 | 1 |  |  |  |  |
|  | ∑ |  | 55 | 41 | 23 | 14 | 9 | 7 | 5 | 4 | 4 | 2 |
|  | negative | MFS | 23 | 15 | 11 | 7 | 5 | 4 | 4 | 3 | 3 | 1 |
|  | positive |  | 12 | 7 | 5 | 1 |  |  |  |  |  |  |
|  | ∑ |  | 35 | 22 | 16 | 7 | 5 | 4 | 4 | 3 | 3 | 1 |
| CD147+ | negative | OS | 10 | 6 | 4 | 3 | 3 | 1 | 1 | 1 | 1 |  |
|  | positive |  | 54 | 43 | 28 | 18 | 13 | 10 | 6 | 5 | 3 | 2 |
|  | ∑ |  | 64 | 49 | 32 | 21 | 16 | 11 | 7 | 6 | 4 | 2 |
|  | negative | TSS | 10 | 6 | 4 | 3 | 2 | 1 | 1 | 1 | 1 |  |
|  | positive |  | 53 | 42 | 27 | 18 | 13 | 10 | 6 | 5 | 3 | 2 |
|  | ∑ |  | 63 | 48 | 31 | 21 | 15 | 11 | 7 | 6 | 4 | 2 |
|  | negative | MFS | 7 | 5 | 4 | 2 | 2 | 1 | 1 | 1 | 1 |  |
|  | positive |  | 32 | 22 | 14 | 7 | 5 | 5 | 4 | 3 | 2 | 1 |
|  | ∑ |  | 39 | 27 | 18 | 9 | 7 | 6 | 5 | 4 | 3 | 1 |
| CD147+CD15+ | negative | OS | 22 | 17 | 12 | 9 | 7 | 6 | 4 | 3 | 3 | 1 |
|  | positive |  | 28 | 22 | 13 | 7 | 4 | 3 | 1 | 1 | 1 | 1 |
|  | ∑ |  | 50 | 39 | 25 | 16 | 18 | 9 | 5 | 4 | 4 | 2 |
|  | negative | TSS | 22 | 17 | 12 | 9 | 7 | 6 | 4 | 3 | 3 | 1 |
|  | positive |  | 28 | 22 | 13 | 7 | 4 | 3 | 1 | 1 | 1 | 1 |
|  | ∑ |  | 50 | 39 | 25 | 16 | 11 | 9 | 5 | 4 | 4 | 2 |
|  | negative | MFS | 14 | 10 | 6 | 4 | 3 | 3 | 3 | 3 | 3 | 1 |
|  | positive |  | 15 | 10 | 7 | 2 |  |  |  |  |  |  |
|  | ∑ |  | 29 | 20 | 13 | 6 | 3 | 3 | 3 | 3 | 3 | 1 |
| HPV+CD147+ | negative | OS | 44 | 33 | 22 | 15 | 12 | 8 | 6 | 5 | 4 | 2 |
|  | positive |  | 21 | 16 | 10 | 6 | 4 | 3 | 1 | 1 |  |  |
|  | ∑ |  | 65 | 49 | 32 | 21 | 16 | 11 | 7 | 6 | 4 | 2 |
|  | negative | TSS | 44 | 33 | 22 | 15 | 11 | 8 | 6 | 5 | 4 | 2 |
|  | positive |  | 20 | 16 | 9 | 6 | 4 | 3 | 1 | 1 |  |  |
|  | ∑ |  | 64 | 49 | 31 | 21 | 15 | 11 | 7 | 6 | 4 | 2 |
|  | negative | MFS | 25 | 19 | 13 | 8 | 6 | 5 | 4 | 4 | 3 | 1 |
|  | positive |  | 14 | 8 | 6 | 2 | 1 | 1 | 1 |  |  |  |
|  | ∑ |  | 39 | 27 | 19 | 10 | 7 | 6 | 5 | 4 | 3 | 1 |
| HPV+CD147+ CD15+ | negative | OS | 48 | 35 | 23 | 16 | 12 | 9 | 7 | 6 | 4 | 2 |
|  | positive |  | 13 | 11 | 7 | 5 | 3 | 2 |  |  |  |  |
|  | ∑ |  | 61 | 46 | 30 | 21 | 15 | 11 | 7 | 6 | 4 | 2 |
|  | negative | TSS | 48 | 35 | 23 | 16 | 11 | 9 | 7 | 6 | 4 | 2 |
|  | positive |  | 13 | 11 | 7 | 5 | 3 | 2 |  |  |  |  |
|  | ∑ |  | 61 | 46 | 30 | 21 | 14 | 11 | 7 | 6 | 4 | 2 |
|  | negative | MFS | 27 | 19 | 13 | 8 | 6 | 5 | 5 | 4 | 3 | 1 |
|  | positive |  | 10 | 6 | 4 | 1 |  |  |  |  |  |  |
|  | ∑ |  | 37 | 25 | 17 | 9 | 6 | 5 | 5 | 4 | 3 | 1 |
| HPV+CD147+ CD15+DKK1+ | negative | OS | 35 | 27 | 17 | 10 | 7 | 5 | 5 | 4 | 4 | 2 |
|  | positive |  | 12 | 10 | 6 | 4 | 2 | 1 |  |  |  |  |
|  | ∑ |  | 47 | 37 | 23 | 14 | 9 | 6 | 5 | 4 | 4 | 2 |
|  | negative | TSS | 35 | 27 | 17 | 10 | 7 | 5 | 5 | 4 | 4 | 2 |
|  | positive |  | 12 | 10 | 6 | 4 | 2 | 1 |  |  |  |  |
|  | ∑ |  | 47 | 37 | 23 | 14 | 9 | 6 | 5 | 4 | 4 | 2 |
|  | negative | MFS | 19 | 15 | 11 | 6 | 4 | 3 | 3 | 3 | 3 | 1 |
|  | positive |  | 9 | 6 | 4 | 1 |  |  |  |  |  |  |
|  | ∑ |  | 28 | 21 | 15 | 7 | 4 | 3 | 3 | 3 | 3 | 1 |
| HPV+CD147+ CD15+p63+ | negative | OS | 38 | 29 | 19 | 12 | 9 | 7 | 5 | 4 | 4 | 2 |
|  | positive |  | 13 | 11 | 7 | 5 | 3 | 2 |  |  |  |  |
|  | ∑ |  | 51 | 40 | 26 | 19 | 12 | 9 | 5 | 4 | 4 | 2 |
|  | negative | TSS | 38 | 29 | 19 | 12 | 9 | 7 | 5 | 4 | 4 | 2 |
|  | positive |  | 13 | 11 | 7 | 5 | 3 | 2 |  |  |  |  |
|  | ∑ |  | 51 | 40 | 26 | 17 | 12 | 9 | 5 | 4 | 4 | 2 |
|  | negative | MFS | 20 | 15 | 10 | 5 | 3 | 3 | 3 | 3 | 3 | 1 |
|  | positive |  | 10 | 6 | 4 | 1 |  |  |  |  |  |  |
|  | ∑ |  | 30 | 21 | 14 | 6 | 3 | 3 | 3 | 3 | 3 | 1 |
| HPV+CD147+ CD15+p63+DKK1+ | negative | OS | 34 | 26 | 16 | 9 | 7 | 5 | 5 | 4 | 4 | 2 |
|  | positive |  | 12 | 10 | 6 | 4 | 2 | 1 |  |  |  |  |
|  | ∑ |  | 46 | 36 | 22 | 13 | 9 | 6 | 5 | 4 | 4 | 2 |
|  | negative | TSS | 34 | 26 | 16 | 9 | 6 | 5 | 5 | 4 | 4 | 2 |
|  | positive |  | 12 | 10 | 6 | 4 | 2 | 1 |  |  |  |  |
|  | ∑ |  | 46 | 36 | 22 | 13 | 8 | 6 | 5 | 4 | 4 | 2 |
|  | negative | MFS | 18 | 14 | 10 | 5 | 3 | 3 | 3 | 3 | 3 | 1 |
|  | positive |  | 9 | 6 | 4 | 1 |  |  |  |  |  |  |
|  | ∑ |  | 27 | 20 | 14 | 6 | 3 | 3 | 3 | 3 | 3 | 1 |
| Nodal status (N) | negative | OS | 96 | 76 | 61 | 45 | 36 | 25 | 12 | 8 | 7 | 3 |
|  | positive |  | 41 | 28 | 14 | 9 | 7 | 5 | 3 | 3 | 2 | 1 |
|  | ∑ |  | 137 | 104 | 75 | 54 | 43 | 30 | 15 | 11 | 9 | 4 |
|  | negative | TSS | 94 | 75 | 60 | 45 | 35 | 23 | 12 | 8 | 7 | 3 |
|  | positive |  | 41 | 27 | 13 | 9 | 7 | 5 | 3 | 3 | 2 | 1 |
|  | ∑ |  | 135 | 102 | 73 | 54 | 42 | 28 | 15 | 11 | 9 | 4 |
|  | negative | MFS | 67 | 52 | 41 | 31 | 27 | 19 | 10 | 7 | 6 | 3 |
|  | positive |  | 27 | 14 | 9 | 5 | 4 | 3 | 2 | 1 | 1 | 1 |
|  | ∑ |  | 94 | 66 | 50 | 36 | 31 | 21 | 12 | 8 | 7 | 3 |
| Invasion status (I) | negative | OS | 45 | 35 | 26 | 19 | 17 | 14 | 8 | 7 | 5 | 2 |
|  | positive |  | 92 | 70 | 49 | 36 | 27 | 17 | 8 | 6 | 5 | 3 |
|  | ∑ |  | 137 | 105 | 75 | 57 | 44 | 31 | 16 | 13 | 10 | 5 |
|  | negative | TSS | 45 | 34 | 26 | 19 | 17 | 13 | 8 | 7 | 5 | 2 |
|  | positive |  | 91 | 69 | 49 | 36 | 26 | 16 | 8 | 6 | 5 | 3 |
|  | ∑ |  | 136 | 103 | 75 | 57 | 43 | 29 | 16 | 13 | 10 | 5 |
|  | negative | MFS | 29 | 21 | 14 | 11 | 11 | 10 | 6 | 5 | 4 | 2 |
|  | positive |  | 65 | 46 | 37 | 26 | 21 | 13 | 7 | 5 | 4 | 2 |
|  | ∑ |  | 94 | 67 | 51 | 37 | 33 | 23 | 13 | 10 | 8 | 4 |
| Vascular invasion (V) | negative | OS | 114 | 88 | 63 | 45 | 36 | 26 | 14 | 10 | 8 | 4 |
|  | positive |  | 24 | 16 | 8 | 6 | 6 | 2 | 1 | 1 |  |  |
|  | ∑ |  | 138 | 104 | 71 | 51 | 42 | 28 | 15 | 11 | 8 | 4 |
|  | negative | TSS | 113 | 86 | 62 | 45 | 35 | 24 | 14 | 10 | 8 | 4 |
|  | positive |  | 24 | 15 | 8 | 6 | 5 | 2 | 1 | 1 |  |  |
|  | ∑ |  | 137 | 101 | 70 | 51 | 40 | 26 | 15 | 11 | 8 | 4 |
|  | negative | MFS | 79 | 58 | 43 | 31 | 26 | 19 | 11 | 8 | 7 | 3 |
|  | positive |  | 15 | 8 | 6 | 4 | 4 | 2 | 1 | 1 |  |  |
|  | ∑ |  | 94 | 66 | 49 | 35 | 30 | 21 | 12 | 9 | 7 | 3 |
| Lymphovascular invasion (L) | negative | OS | 122 | 92 | 66 | 48 | 38 | 27 | 15 | 11 | 8 | 4 |
|  | positive |  | 16 | 11 | 4 | 2 | 2 | 1 |  |  |  |  |
|  | ∑ |  | 138 | 103 | 70 | 50 | 40 | 28 | 15 | 11 | 8 | 4 |
|  | negative | TSS | 121 | 90 | 65 | 48 | 38 | 25 | 15 | 11 | 8 | 4 |
|  | positive |  | 16 | 10 | 4 | 2 | 2 | 1 |  |  |  |  |
|  | ∑ |  | 137 | 100 | 69 | 50 | 40 | 26 | 15 | 11 | 8 | 4 |
|  | negative | MFS | 82 | 60 | 45 | 33 | 28 | 20 | 12 | 9 | 7 | 3 |
|  | positive |  | 12 | 6 | 3 | 1 | 1 | 1 |  |  |  |  |
|  | ∑ |  | 94 | 66 | 48 | 34 | 29 | 21 | 12 | 9 | 7 | 3 |
| Perineural invasion (Pn) | negative | OS | 83 | 67 | 49 | 33 | 25 | 16 | 8 | 5 | 3 | 2 |
|  | positive |  | 11 | 6 | 2 | 1 | 1 |  |  |  |  |  |
|  | ∑ |  | 94 | 73 | 51 | 34 | 26 | 16 | 8 | 5 | 3 | 2 |
|  | negative | TSS | 82 | 66 | 48 | 33 | 24 | 15 | 8 | 5 | 3 | 2 |
|  | positive |  | 11 | 6 | 2 | 1 | 1 |  |  |  |  |  |
|  | ∑ |  | 93 | 72 | 50 | 34 | 25 | 15 | 8 | 5 | 3 | 2 |
|  | negative | MFS | 52 | 38 | 29 | 18 | 14 | 10 | 6 | 3 | 2 | 1 |
|  | positive |  | 8 | 3 | 1 | 1 | 1 |  |  |  |  |  |
|  | ∑ |  | 60 | 41 | 30 | 19 | 15 | 10 | 6 | 3 | 2 | 1 |

**Supplementary Figure 9: Representative IHC staining of biomarkers**

**
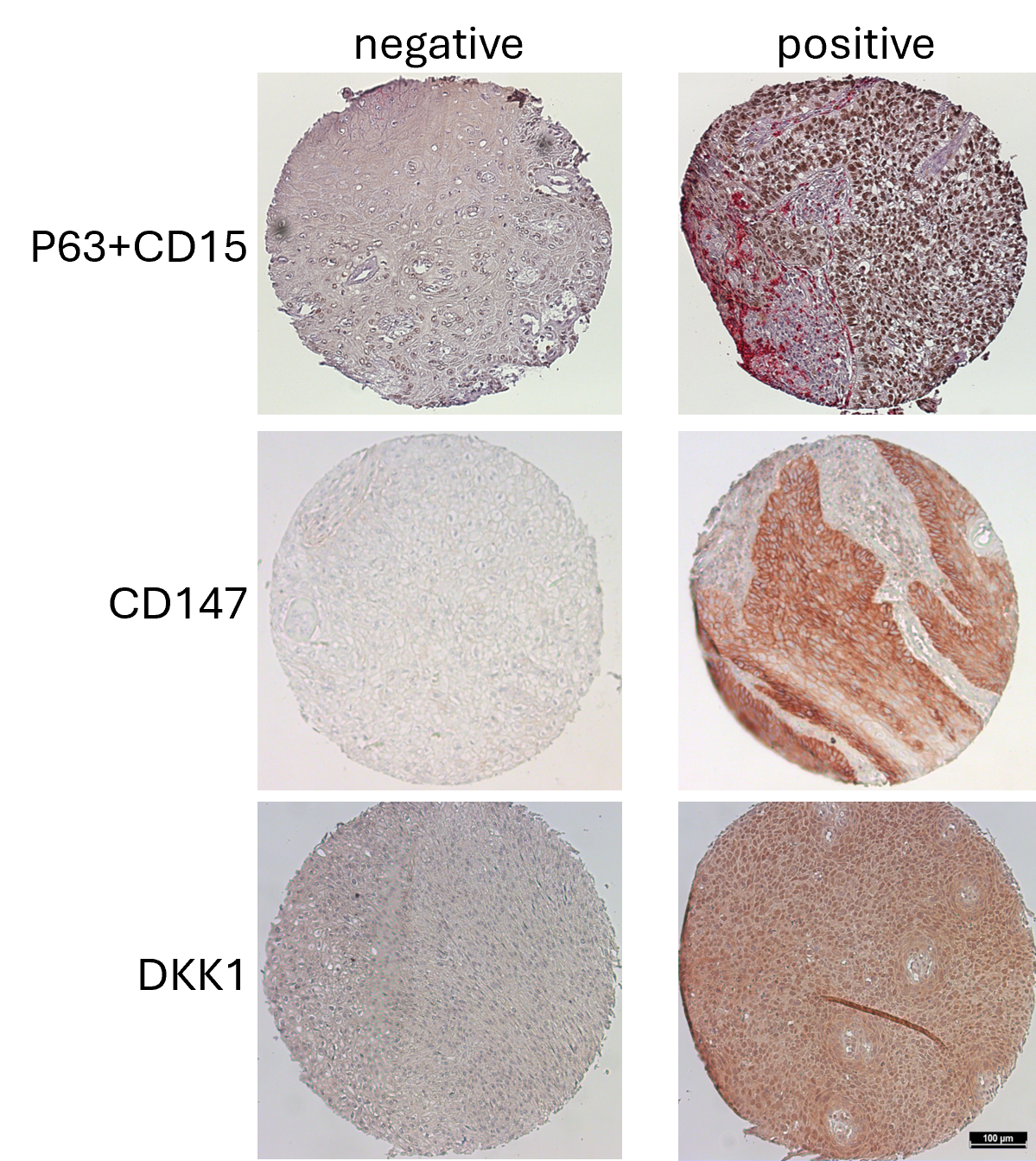
**
